# Domain-specific functional coupling between dorsal and ventral systems during action perception

**DOI:** 10.1038/s41598-020-78276-4

**Published:** 2020-12-03

**Authors:** Huichao Yang, Chenxi He, Zaizhu Han, Yanchao Bi

**Affiliations:** 1 State Key Laboratory of Cognitive Neuroscience and Learning & IDG/McGovern Institute for Brain Research, Beijing Normal University, Beijing, 100875 China; 2 Beijing Key Laboratory of Brain Imaging and Connectomics, Beijing Normal University, Beijing, China

**Keywords:** Neuroscience, Cognitive neuroscience, Perception, Psychology, Human behaviour

## Abstract

Visual perception of actions and objects has been shown to activate different cortical systems: action perception system spanning more dorsally, across parietal, frontal, and dorsal temporal regions; object perception relying more strongly the ventral occipitotemporal cortex (VOTC). Compared to the well-established object-domain structure (e.g., faces vs. artifacts) in VOTC, it is less known whether the action perception system is constrained by similar domain principle and whether it communicates with the ventral object recognition system in a domain-specific manner. In a fMRI long-block experiment designed to evaluate both regional activity and task-based functional connectivity (FC) patterns, participants viewed animated videos of a human performing two domains of actions to the same set of meaningless shapes without object-domain information: social-communicative-actions (e.g., waving) and manipulation-actions (e.g., folding). We observed action-domain-specific activations, with the superior temporal sulcus and the right precentral region responding more strongly during social-communicative-action perception; the supramarginal gyrus, inferior and superior parietal lobe, and precentral gyrus during manipulation-action perception. The two domains of action perception systems communicated with VOTC in domain-specific manners: FC between the social-communicative-action system and the bilateral fusiform face area was enhanced during social-communicative-action perception; FC between the manipulation-action system and the left tool-preferring lateral occipitoptemporal cortex was enhanced during manipulation-action perception. There was a significant correlation between the FC-with-action-system and the local activity strength across VOTC voxels. Our findings highlight social- and manipulation-domains of human interaction as an overarching principle of both object and action perception systems, with domain-based functional communication across systems.

## Introduction

Visual perception of actions and objects has been shown to activate different cortical systems. Object recognition primarily relies on the ventral occipitotemporal cortex (VOTC)^1,2^; Action observation activates predominantly the occipitoparietal region, posterior dorsal temporal gyrus and the inferior frontal gyrus (IFG)^3,4^. VOTC has a well-established object “domain” organization, with different patches sensitive to several major, broad, evolutionarily-salient domains of objects (e.g., faces, animals, scenes and navigation-related large objects, small manipulable objects, with a broad animate/inanimate domain distinction)^5–7^. Although the interaction between the visual ventral and dorsal pathways (what vs. how/where)^8,9^, which overlapped with part of the action perception network, has been well documented^10–18^, the evidence about whether action perception and its communication with the ventral object regions is also guided by parallel processing-domain-structure (social purpose vs. object purpose) is less clear-cut.

Several lines of evidence have suggested a domain-related interaction pattern between object and action perception systems. Different domains of objects activate dorsal regions that are implicated in action perception: Manipulable objects activated the inferior parietal lobe (IPL)^19^ supporting manipulation knowledge of tools and hand-object action perception^3,4,20–22^; faces and animals activated the posterior superior temporal sulcus (pSTS)^23^ related to biological motion and social interaction perception^4,22,24,25^. The structural or resting-state functional brain connectivity of ventral-object and dorsal-action systems were also organized into a domain-like pattern: tool-preferring ventral areas were connected with the frontoparietal hand-arm/manipulation action processing regions^13,26–29^, and face-preferring ventral areas with the pSTS social cognition region^30^. Functional connectivity (FC) between tool-preferring ventral and dorsal regions was enhanced during action-performing tasks^12,14^. Recent studies have further shown the causal influence of the left inferior parietal areas on the ventral regions or object representations: lesions or stimulation to this region modulate the tool representations in the ventral-medial tool-preferring areas^31,32^.

These findings are only indirect evidence for whether action perception system itself is organized by “domains” and how it communicates with the ventral object system dynamically. Given that the studies reviewed above tended to involve object stimuli (e.g., object names, movies of real multi-object contexts, but see Centelles et al.^24^), it is possible that these effects were driven by object-domain properties (e.g., animate/biological vs. artifact object). Objects are recognized by the ventral visual system, which is organized by the salient object domains (i.e., animate/inanimate), and activate the typical action knowledge about that object stored in the dorsal system through brain connections, or activate the parietal regions directly through subcortical pathways^33,34^. It is thus not clear whether domains of actions are only organized along the animate/inanimate dimensions (a car moving vs. a person walking), or by domains that are beyond object properties, such as social-communicative-actions versus manipulation-actions^35^. For instance, pSTS has been robustly implicated in biological motion^36–39^, but it is unknown whether this region has different degree of sensitivities to different kinds of biological motion (social- vs. manipulaiton-actions). Note that Centelles et al.^24^ have tried to exclude the effects of object properties and found stronger pSTS activations when participants watching two individuals interacting compared with two individuals acting independently using point-light stimuli. This comparison was between human-social-goal-directed versus non-goal-directed movements, whether pSTS differentiate different goal-directed biological motions (human-directed social-communicative-actions vs. object-directed manipulation-actions) is unknown. Also unclear is whether action perception entails dynamic functional communications between the dorsal action perception network with the ventral object processing stream in a domain-specific manner. Here, we test the hypothesis of a domain-organized action perception pathway using action stimuli excluding object domain differences, with an experiment designed to optimally evaluate both regional activities and task-based FC patterns. Participants watched videos of a human cartoon figure performing two types of actions (social-communicative-actions such as waving, manipulation-actions such as folding) to a same set of meaningless shapes during fMRI scanning. We examined whether the two action-perception conditions elicited different dorsal action perception system activations and whether such activations communicated with the ventral system differently.

## Results

### Identifying the domain-specific action perception systems: Social-communicative-actions versus manipulation-actions

First, both types of action perception conditions, compared to baseline, activated the bilateral IFG, superior parietal gyri, and posterior superior to inferior temporal gyri (voxel level *p* < 0.0001, cluster-extent FWE *p* < 0.05; Supplementary Fig. S1). We then carried out whole-brain univariate contrasts between conditions where participants watched videos of social-communicative-actions and manipulation-actions (voxel level *p* < 0.0001, cluster-extent FWE *p* < 0.05).

#### Social-communicative-actions video-watching

**Figure 1:**
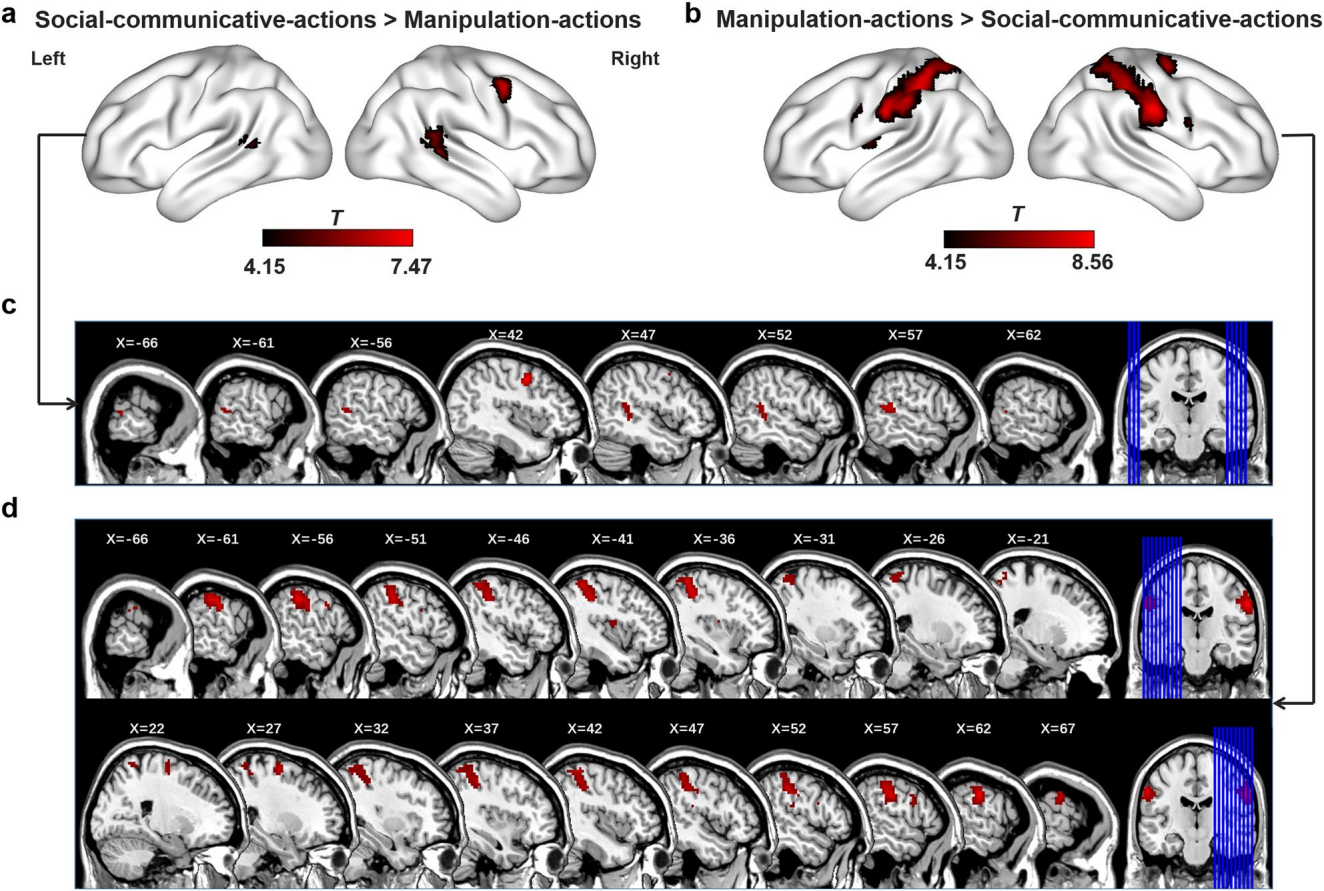
Whole brain univariate analysis results of social- and manipulation-action perception. **a**,**c** Cortical surface and multi-slice presentation of social-communicative-action specific activation relative to the manipulation-action condition. **b**,**d** Cortical surface and multi-slice presentation manipulation-action specific activation relative to the social-communicative-action condition. Threshold: voxel level *p* < 0.0001, cluster-extent FWE corrected *p* < 0.05.

Social-communicative-actions, simulating human–human interaction such as waving, induced greater activation than manipulation-actions in the right precentral gyri (Prec), bilateral pSTS/posterior middle temporal gyrus (pMTG; Fig. 1a,c and Table 1).

**Table 1:** Whole-brain univariate analysis results of social- or manipulation-action-specific activations.

Contrast	Anatomical regions of the cluster's peak voxel (other including regions)	MNI coordinates of peak voxel (mm)			*t*	Cluster size
		x	y	z		
SA versus MA	Right Prec	42	3	45	7.47	43
	Right STG (MTG)	57	− 42	15	5.73	71
	Left MTG	− 66	− 42	9	5.09	20
MA versus SA	Right Posc (IPL; SMG; SPL)	60	− 18	33	8.56	459
	Left SPL (Posc; IPL, SMG)	− 36	− 45	60	8.17	462
	Right SFG (Prec)	27	− 9	63	6.80	52
	Left Prec	− 54	6	36	5.76	15
	Right IFoper (Prec)	54	9	24	5.46	21
	Left insular (rolandic oper)	− 39	− 6	12	5.10	16

Threshold: voxel level *p* < 0.0001, cluster-extent FWE corrected *p* < 0.05.

*SA* social-communicative-actions, *MA* manipulation-actions, *Prec* precentral gyrus, *STG* superior temporal gyrus, *MTG* middle temporal gyrus, *Posc* postcentral gyrus, *IPL* inferior parietal lobe, *SMG* supramargical gyrus, *SPL* superior parietal lobe, *SFG* superior frontal gyrus, *IFoper* inferior frontal operculum.

#### Manipulation-actions video-watching

Manipulation-actions (e.g., folding an object) induced greater activation than social-communicative-actions in the bilateral supramarginal gyri (SMG), bilateral IPL, bilateral superior parietal lobe (SPL), bilateral postcentral gyri (Posc), bilateral Prec, right superior and inferior frontal gyri, and left insula (Fig. 1b,d and Table 1).

**Figure 2:**
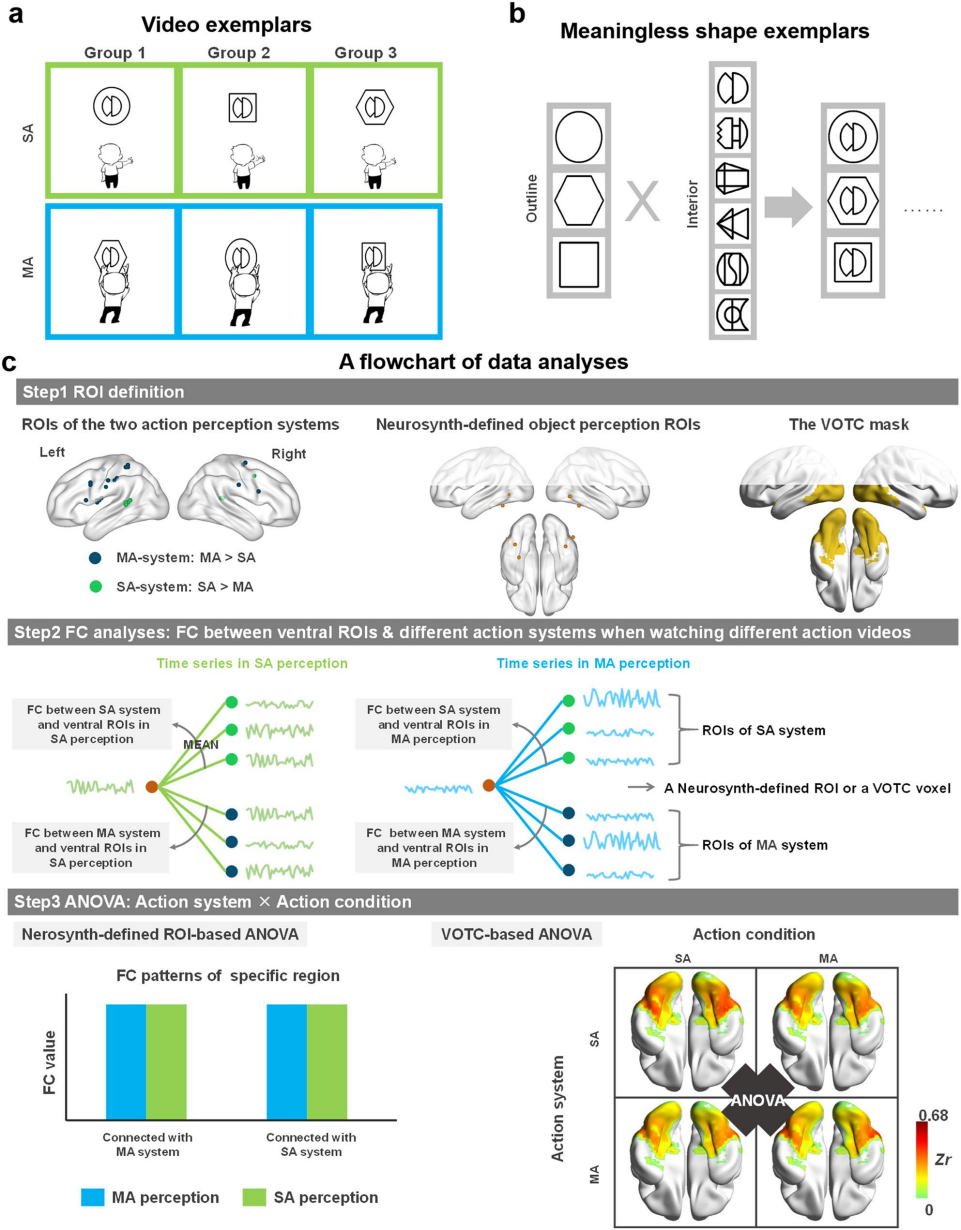
Examples of stimuli and analysis flowchart. **a** Examples of video stimuli. Three participant groups with equal number watched videos of three different action-shape correspondence rules to make meaningless shapes fully matched at the group level. **b** Examples of meaningless shapes used in the videos. Left panel, the outline shapes; Middle panel, the interior meaningless shapes; right panel, combined meaningless shape exemplars. All video stimuli were shown in Supplemental files. **c** Flowchart of FC analyses. Step 1 was ROI definition: the manipulation- or social-communicative-action system were defined by the two types of action perception comparison maps during video-watching using leave-one-participant out methods, the classical ventral object perception ROIs were defined by Neurosynth meta-analyses and the VOTC mask was defined from our previous study^57^. Step 2 was to compute FCs with social- or manipulation-action systems under different task conditions for each Neurosynth-defined ROI or each VOTC voxel. Different colored curves represent time series extraction in different conditions of the ROIs: light blue = manipulation-action perception; light green = social-communicative-action perception. Step 3 was an ANOVA of the four FC measures (the two action systems × two action conditions). *MA* manipulation-actions, *SA* social-communicative-actions.

These results indicate that social-communicative-actions and manipulation-actions, without object-domain information (same set of human cartoon figure and arbitrary meaningless shapes), elicited different distributed activations across frontal, parietal and dorsal temporal regions. Worth-noting is that both the social-communicative-actions and the manipulation-actions activated bilateral pSTS relative to baseline (Supplementary Fig. S1), in line with previous findings that highlighted the role of pSTS in biological motions^36–39^. Importantly here, the pSTS showed stronger sensitivity to social-communicative-action perception, indicating effects beyond biological motion per se. Also note that even though the cartoon figure and meaningless shapes may have some form changes during the actions (e.g., Fig. 2a, the form of the shape changes when the cartoon figure acts on it in the manipulation-action condition), these form changes do not correspond to the object domain differences. They did, however, result in more cumulative movement information in this condition than the social-communicative-actions (see “Experimental design” section). To examine if any results for the manipulation action were simply due to sensitivity to more visual changes (movements), we looked at the navigation condition that were not of interest in the current study, which had even higher cumulative movements than the manipulation-actions (*p* = 1.185 × 10^–29^). We compared the activation strengths in those manipulation specific-activation regions between navigation condition and the manipulation-actions, with the rationale that if these regions simply responded to more actions, they should show higher responses to navigation than to manipulation actions. All but one of the manipulation specific-activation clusters showed higher activations for the manipulation-actions or no differences with the navigation, indicating that the manipulation-action-specific effects in these regions were not simply attributable to more movements in video stimuli (Supplementary Fig. S5a). The one exception located in the right IFG/Prec (MNI peak coordinates: 54, 9, 24), showing stronger activation in the navigation than in the manipulation-actions. We thus performed additional validation analyses excluding this cluster in the FC analyses (ROI-based) below, and the results were fully replicated (Supplementary Fig. S5b–e).

### FC analyses: communication pattern between the domain-specific action perception system and the ventral object perception regions

To explore whether and how the action perception system communicates with the ventral object perception regions in a domain-specific manner, we calculated the FC strength between the two action systems obtained for each participant (see “Methods” section) and VOTC in different task conditions. That is, for each Neurosynth-defined object domain ROI (in the ROI analysis) or each VOTC voxel (in the whole VOTC mask analysis) we obtained four FC measures: FC strength with the two action systems (social-communicative-action and manipulation-action) in the two video conditions (social-communicative-action perception; manipulation-action perception) and applied a 2 × 2 repeated-measure ANOVA (Fig. 2c). Only those results showing statistical significance are reported below.

#### ROI analysis results

**Figure 3:**
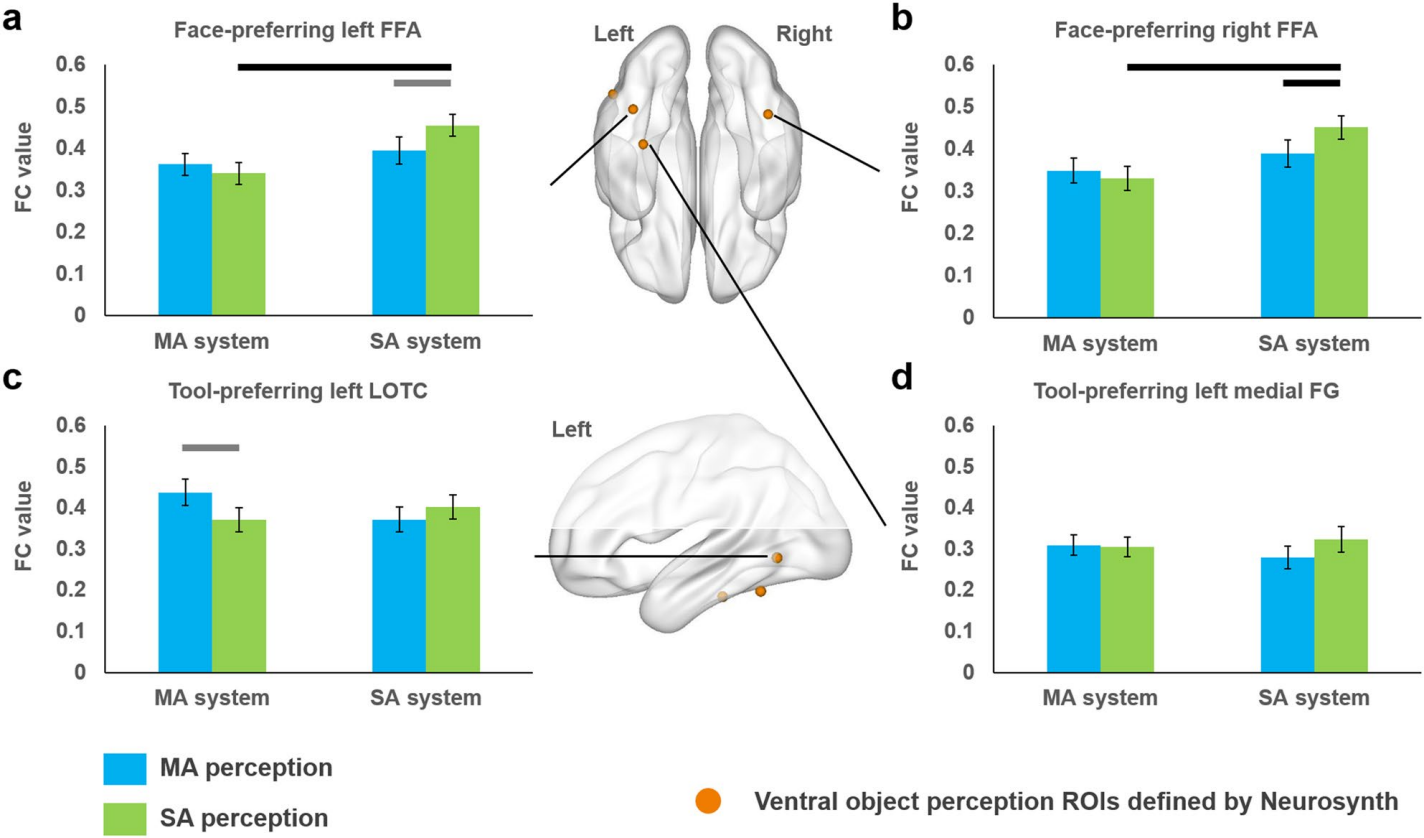
Results of Neurosynth-defined ventral ROI-based ANOVA. **a**,**b** FC patterns of the face-preferring face fusiform area (FFA): Enhanced connection with the social-communicative-action system in social-communicative-action perception. **c** FC patterns of the tool-preferring left lateral occipitotemporal cortex (LOTC): Enhanced connection with the manipulation-action system in manipulation-action perception. **d** FC patterns of the tool-preferring left medial fusiform gyrus (medFG): no interaction effects. We applied tests of simple effects either within the same action system or within the same action condition for the Neurosynth-defined ventral object perception ROIs showing significant interaction effects; lines above bars indicate significant difference between the two bars (black: adjusted *p* < 0.05; gray: uncorrected *p* < 0.05). *MA* manipulation-actions, *SA* social-communicative-actions.

To examine whether action perception system connected with ventral object perception regions in a domain-specific manner from a theory-driven perspective, analyses were carried out on VOTC ROIs showing face- or tool-preferring activations, defined by Neurosynth meta-analyses (Fig. 3, Supplementary Table S4; see also Methods). Face preferring ROI (bilateral FFA) and tool preferring ROI (left LOTC) showed significant interaction effects between action perception system and action viewing conditions (left FFA, *F*(35) = 7.699, *p* = 0.009; right FFA, *F*(35) = 8.773, *p* = 0.005; left LOTC, *F*(35) = 11.581, *p* = 0.002). Tests of simple effects revealed that FFA significantly increased their FCs with the social-communicative-action system during social-communicative-action perception condition compared to the manipulation-action perception condition, with the right FFA reaching corrected threshold (green bar > blue bar for social-communicative-action system in Fig. 3a,b; left: *t*(35) = 2.474, uncorrected *p* = 0.018; right: *t*(35) = 2.737, adjusted *p* = 0.039). Their connection with the social-communicative-action system was also significantly stronger than with the manipulation-action system in the social-communicative-action perception condition (two green bars in Fig. 3a,b; left: *t*(35) = 4.878, adjusted *p* = 9.259 × 10^–5^; right: *t*(35) = 5.235, adjusted *p* = 3.147 × 10^–5^). The left LOTC showed marginally stronger connections with the manipulation-action system in the manipulation-action perception condition than in the social-communicative-action perception condition (Fig. 3c; *t*(35) = 2.348, uncorrected *p* = 0.025). Neither main effects nor interaction effects were found for the left medFG tool-preferring ROI (*p*s ≥ 0.086; Fig. 3d). As previously mentioned, the right IFG/Prec cluster from the manipulation-action perception system has potential sensitivity to the amount of movement (Supplementary Fig. S5a). We thus excluded this cluster to replicate the ROI analyses and obtained the same pattern of results (Figure S5b–e).

#### Whole VOTC mask analysis results

**Figure 4:**
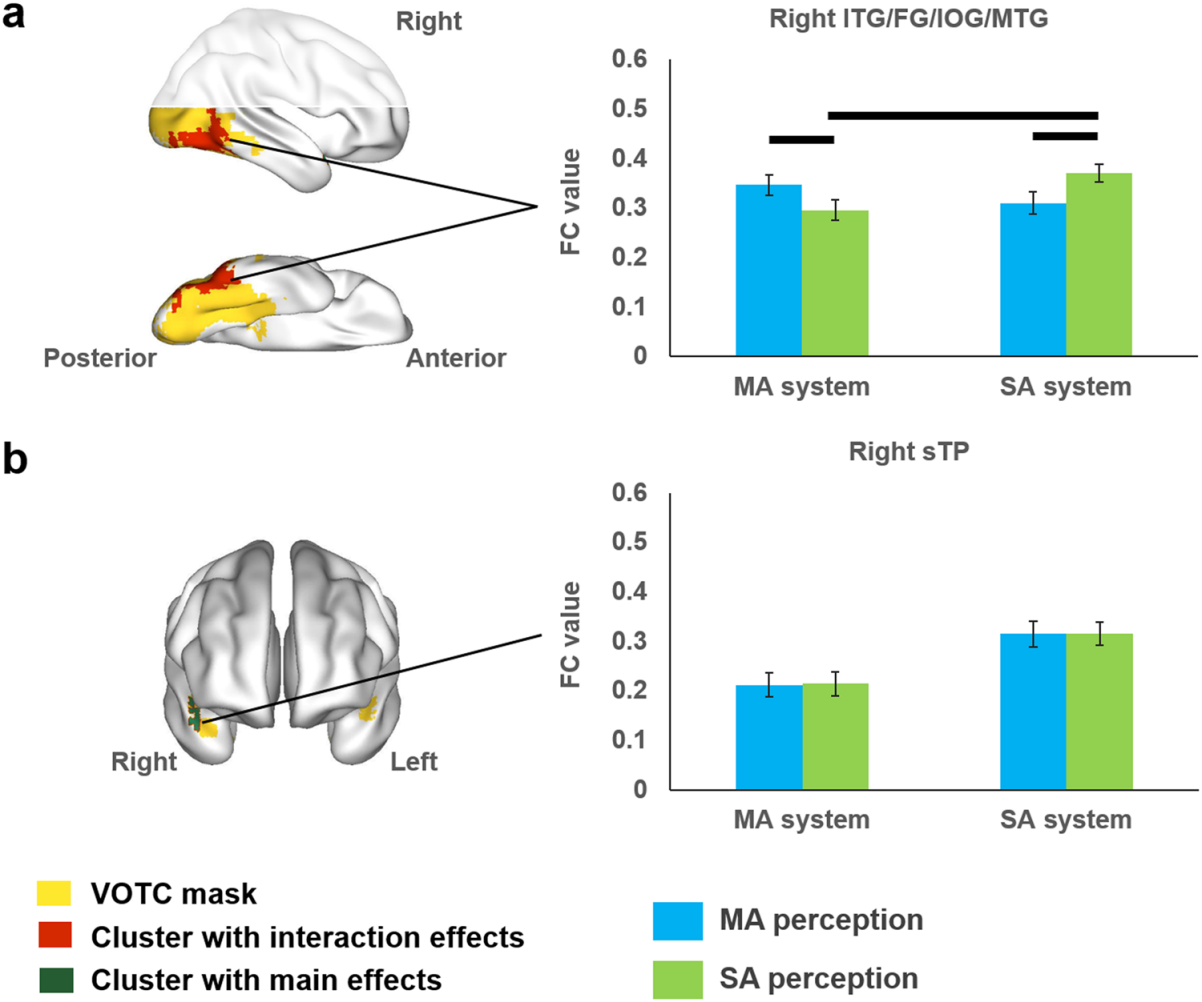
Results of VOTC-based ANOVA. **a** The right ITG/FG/IOG/MTG cluster showed significant interaction effects between action system and action condition (red; threshold: voxel level *p* < 0.001, cluster-extent FWE *p* < 0.05). We applied tests of simple effects either within the same action system or within the same action condition; black lines above bars indicate significant difference between the two bars (adjusted *p* < 0.05). **b** The right sTP cluster showed significant main effect of action system without interaction with action conditions (green; threshold: voxel level at *p* < 0.001, cluster-extent FWE corrected *p* < 0.05). *ITG* inferior temporal gyrus, *FG* fusiform gyrus, *IOG* inferior occipital gyrus, *MTG* middle temporal gyrus, *sTP* superior temporal pole. Note: only clusters significant in both primary and validation analyses (i.e., using data without and with global signal regression) were reported; others are shown in the Supplementary Figs. S7 and S8. *MA* manipulation-actions, *SA* social-communicative-actions.

We further checked the whole VOTC mask in a voxel-wise manner. Given the data-driven nature of this analysis, we presented those clusters significant (voxel level *p* < 0.001, cluster-extent FWE *p* < 0.05; either main or interaction effects) in both primary and validation analyses (i.e., with or without global signal regression) as the main results to maximize robustness. Full sets of results for each analysis separately are shown in the Supplementary Figs. S7, S8, Tables S2, S3. One cluster encompassing the right inferior temporal gyrus (ITG) and fusiform gyrus (FG) was obtained showing significant interaction (Fig. 4a and Table 2): It connected with the manipulation-action system more strongly in the manipulation-action condition than in the social-communicative-action condition (*t*(35) = 3.308, adjusted *p* = 0.009), and with the social-communicative-action system more strongly in the social-communicative-action condition than in the manipulation-action condition [*t*(35) = 3.636, adjusted *p* = 0.004].

**Table 2:** Results of VOTC-based ANOVA (domain-action system × domain-action condition).

Anatomical regions of the cluster's peak voxel (other including regions)	MNI coordinates of peak voxel (mm)			F	Cluster size
	x	y	z		
**Cluster showing significant interaction effects**					
Right ITG (FG; IOG; MTG)	54	− 66	− 12	32.82	270
**Cluster showing significant main effect of action systems**					
Right sTP	51	18	− 18	54.77	59

Threshold: voxel level *p* < 0.001, cluster-extent FWE corrected *p* < 0.05. Only clusters significant (voxel level *p* < 0.001, cluster-extent FWE *p* < 0.05) in both primary and validation analyses were shown to maximize robustness.

*sTP* superior temporal pole, *ITG* inferior temporal gyrus, *FG* fusiform gyrus, *IOG* inferior occipital gyrus, *MTG* middle temporal gyrus.

A cluster in the right superior temporal pole (sTP) showed a significant main effect of action system without interaction with action condition: it had stronger connections with the social-communicative-action system than with the manipulation-action system (Fig. 4b, Table 2), suggesting intrinsic connection differences with the two systems that were unaffected by the particular action inputs. We noticed that this cluster was closer to the social-communicative-action system than the manipulation-action system (Euclidean distance: 89.142 ± 0.589 vs. 91.746 ± 4.373; *t*(35) = 3.401, *p* = 0.002), and that this FC main effect was no longer significant using residual FCs after regressing out the Euclidean distances with the two action systems. We thus did not consider this cluster further.

### Relationship between VOTC’s FC with dorsal action perception systems and VOTC’s local activation strength

**Figure 5:**
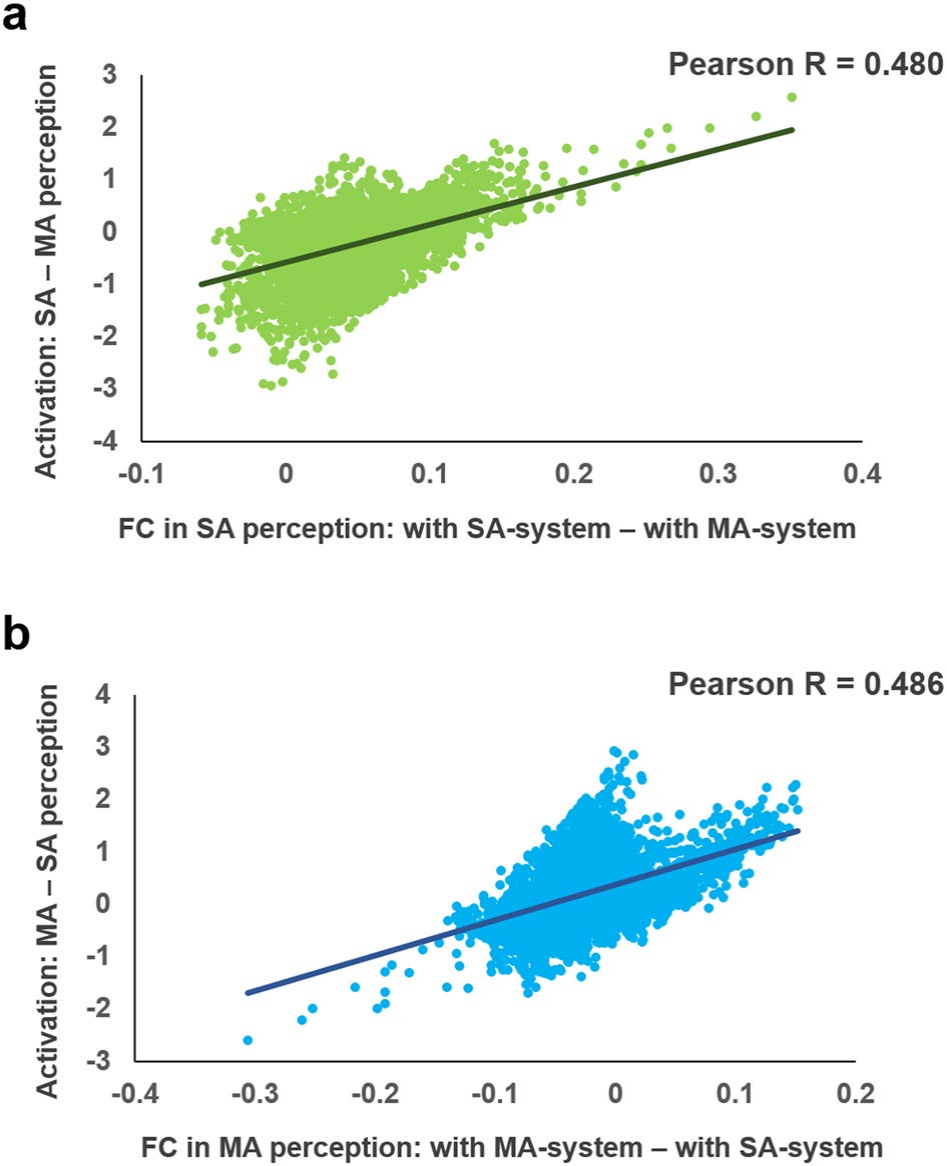
Correlation between the domain-specific FC (connected with one of the action systems relative to the other one in the corresponding action perception condition) and domain-specific local activation strength (in one action condition relative to the other) across VOTC voxels. Correlations for both domains (social vs. manipulation) were significant (*p*s ≤ 5.550 × 10^–225^). Each dot represents a VOTC voxel (*N* = 3915), with green dots for the social-communicative-action domain and blue dots for the manipulation-action domain. *SA* social-communicative-action perception, *MA* manipulation-action perception.

Although we did not observe above-threshold significant effects of domain-specific action perception activations in VOTC, to examine whether the VOTC activation strength is systematically affected by the connections with the dorsal action perception systems, we computed the correlation between the domain-specific FCs with the dorsal action perception systems and domain-specific activation strength across VOTC voxels. The correlation between the relative FC strength (to social-communicative-action system minus to manipulation-action system) during social-communicative-action perception and the relative activation strength (social-communicative-action perception minus manipulation-action perception) was highly significant (Pearson R = 0.48, *p* = 5.550 × 10^–225^, Fig. 5a); Likewise, the correlation between the relative FC strength (to the manipulation-action system minus to the social-communicative-action system) during manipulation-action perception and the relative activation strength (manipulation-action perception minus social-communicative-action perception) was also significant (Pearson R = 0.486, *p* = 3.934 × 10^–231^, Fig. 5b).

## Discussion

**Figure 6:**
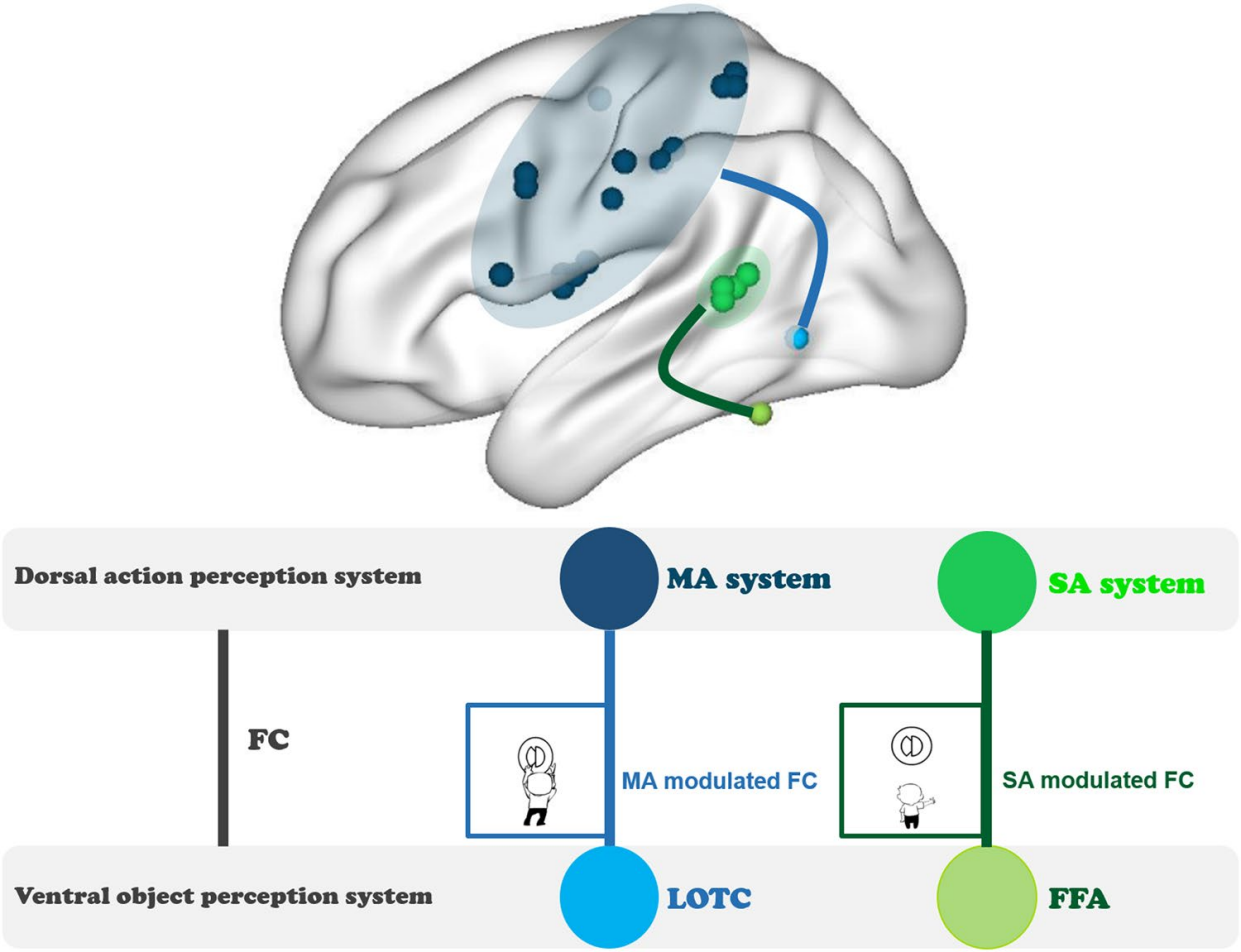
Summary of the FC patterns between the dorsal action perception system and the ventral object perception regions. *MA* manipulation-actions, *SA* social-communicative-actions.

By excluding typical object-domain properties and manipulating human action contents, we tested whether action perception is processed by action domains of social- and manipulation-actions, and how action-perception regions communicate with the ventral visual pathway. There were two main findings. First, perception of social- and manipulation-actions elicits different activations in the parietal, frontal and superior temporal cortex, with social-communicative-actions such as waving activating the bilateral pSTS and right Prec, and manipulation-actions such as folding activating the bilateral SMG, IPL, SPL, Prec and Posc. Second, during action perception, these two systems communicate with the ventral system differently (see summary in Fig. 6), with FC between the social-communicative-action system and the bilateral FFA enhanced during social-communicative-action perception, and FC between the manipulation-action system and left LOTC enhanced during manipulation-action perception. Whole VOTC analyses yielded cluster encompassing the right ITG and FG that showed a tendency to be modulated by both action conditions, with connection with one of the action systems stronger in the corresponding action condition than the other condition. Such action-domain-driven FC patterns converge with the object-domain distribution pattern in VOTC, with a significant correlation between the FC-with-action-system and local activity strength across VOTC voxels. Below we discuss these two findings in turn.

### Action-domain effects in action perception

Our finding that social-communicative-action perception, relative to manipulation-action perception, elicits stronger activation in the bilateral pSTS, is consistent with previous studies of biological motion or communicative actions using real and point-light social-action videos^22,24,25,35,40,41^. The stronger right Prec region activation has been consistently observed in face-movement processing^4^. The effects in this cluster might be because social-communicative-actions are often accompanied with face expressions (e.g., people often smile when they wave to other people), this region might be activated by means of top down processing. For manipulation-action perception, the stronger activation in the SMG/IPL, SPL and Prec, is consistent with previous findings when participants viewed transitive object-directed action videos^22^ and human-object action perception^3,4^ (meta-analyses). As reasoned in the Introduction, in these previous studies the actions were presented along with the entity properties. Our findings, using the same set of human figures and meaningless shapes across social- and manipulation-action conditions, revealed that the sensitivity of these regions to social-communicative-action versus manipulation-action cannot be fully attributed to properties of the corresponding object domains (i.e., animate/biological vs. artifact shape properties), but related to the different types of action patterns or action consequences. Our manipulation-actions, in order to be natural, caused the target to go through a form change as a mechanical consequence of manipulation. Social-communicative-actions do not have the same type of mechanical consequence on the target. The more specific differences between the two domains of action patterns need to be tested in the future^42^. Worth further noting is that pSTS has been proposed to participate in social-action perception by processing biological (articulated) motion^36–39^. However, in our study both action types were biological motion (performed by a human figure), suggesting that the contribution of pSTS to social-communicative-action perception is beyond biological motion perception per se, and may reflect sensitivity to differences between different types of biological motion. Also, the manipulation-action perception regions overlapped greatly with the regions that are activated during tool pantomiming^20^. The exact functional role of these regions in action perception and their relation with action performance remains to be examined^43^.

### Domain-specific dorsal–ventral communication patterns and relation with the ventral activity patterns

Regarding how the dorsal action perception system communicates with the ventral visual system, here we observed differences by domain. The social-communicative-action perception system enhanced its connection with bilateral FFA during viewing of social-communicative-actions; the manipulation-action system enhanced its connections with left tool-preferring LOTC during viewing of manipulation-actions. Note that the functional connectivity results could not be simply attributed to the activation differences in the dorsal action perception system (e.g., signal-to-noise differences) because we actually observed significant interactions between these two measures. It has recently been shown that during reaching/grasping or tool-use pantomime tasks, the lateral ventral occipital temporal regions connect with the dorsal frontal-parietal action regions (using tool names as stimuli in Garcea et al.^12^; using real objects in Hutchison and Gallivan^14^). Our results established the domain-specificity beyond this tool-domain (i.e., social- and manipulation-domain-specificity). Our findings also converge partly with the dorsal-to-ventral gradients for human-directed to object-directed actions^35^ by showing that during social-communicative-action perception pSTS is more strongly activated. Although we did not find stronger activation more inferiorly for manipulation (object-directed) action, probably because the pMTG finding in their study was driven by properties of typical objects that were deliberately absent in our study, we did find its enhanced functional connection with the manipulation action system more dorsally. More generally, by excluding typical object-domain differences and contrasting action patterns, our results extend the previous findings by showing that such connection patterns are not fully attributable to specific visual differences between animate and inanimate objects (e.g., rectilinear vs. curvature^44^), and that there is a social versus manipulation domain-correspondence pattern across action and object systems. The domain-specific task-based functional connection patterns we observed are also in line with resting-state FC studies: Peelen et al.^27^ found pMTG connected with the frontal-parietal action network during resting-state; Turk-Browne et al.^30^ found FG face area connected with pSTS regions during resting-state. Our findings show that not only are the ventral-object and dorsal-action domain-preferring regions connected at rest, they are specifically enhanced by task in a domain-specific fashion. Past studies in the tool domain also found stronger FC between the left IPL and medFG compared with other object-preferring VOTC regions during resting-state^29^. Intriguingly here, we did not find stable effects of the left medFG-IPL/SMG in a task-driven, domain-specific manner. In the validation analysis with global signal regression the medFG was found to connect with manipulation-action system more strongly during both action perception conditions (Supplementary Fig. S6d), in line with the resting-state FC findings, but did not hold if global signal regression was not performed. Taken together, it is likely that the medFG is intrinsically connected with IPL, but is not additionally modulated by online action perception tasks, at least without object-related bottom-up information.

Across all VOTC voxels, we found a moderate correlation (rs = 0.480 and 0.486) between the FC-with-action-perception-systems and the local activity strength in a domain-specific fashion. Although our analyses with the fMRI data do not allow for directionality test, we may infer the origin of the effects based on the experimental manipulations. In our experiments the (static) visual properties in the two conditions did not differ along any obvious domain dimensions, the VOTC local activity differences between action perception tasks are thus more likely driven by communications with the action perception process than the reverse. These findings also extend findings of two recent studies, which showed the causal impact of the inferior parietal regions on the activities of the tool-preferring region in VOTC using lesion and stimulation techniques^31,32^. The current results revealed that the neural basis of such influence might be through dynamically modifying the corresponding functional connectivities. That is, in a real-world dynamic visual event involving both action and object information, the activations in the classical ventral object perception regions may be the summed results of two types of inputs: bottom-up properties (i.e., object shape) and through connectivity from the action-perception system (see similar discussions about LOTC in Lingnau & Downing^15^). This observation may also explain the discrepancies between our results and two previous studies. Castelli et al.^45^ compared activation between geometric shapes interacting with each other in a social way (e.g., persuading) to the same shapes with random mechanical motions (e.g., bouncing) during a passive viewing task. In Schultz et al.^46^, the participants were asked to pretend the geometric shapes were people and judge whether they were friends, or to pretend the shapes were bumper cars and judge whether they were the same weight. Both studies found significantly stronger FG activity in social animation using geometric shapes, while we did not find statistically significant effects in VOTC between the two action perception conditions. These active “images” (participants pretended the geometric shapes were people) may supply the internal bottom-up inputs for the ventral system and together with the “action perception” inputs, leading to above-threshold activations. No participants reported pretending the meaningless shapes as people in the post-experimental survey of our study. Most of them named the meaningless shapes according to their shapes (e.g. diamond) and then establish relationship between the actions and shapes through rote learning.

### Conclusions

The current results of social- versus manipulation-action domain organization in the action perceptual system (parietal, frontal, and dorsal temporal cortex), domain-specific functional-connection pattern with the ventral object pathway, together with the classical object domain-organization in the ventral visual pathway (faces in FFA vs. manipulable small objects in LOTC), reflect a unified principle in perception: social- versus manipulation- domains. This is in accord with the general notion of the connectivity-constrained domain representation hypothesis^47^. Previously, “domain” has been used usually in the context of object representation (conspecifics, tools, animals, etc.). Our findings highlight the significance of domains of human-interaction as an overarching principle: Object-manipulation and social-interaction for both ventral and dorsal visual perception system, and demonstrate domain-based dynamic functional communication across systems^48^.

## Methods

### Participants

Forty-four right-handed individuals (20 males; 22.4 ± 2.4 years old, range 18–28 years old) with normal or corrected-to-normal vision participated in this study. Thirty-six (15 males; 22.2 ± 2.4 years old, range 18–28 years old) of them were included in the following analyses. Eight participants were excluded for excessive head-motion and balance of the action-shape matching rules (see the following parts and Supplementary Table S1 for details). Results of using more liberal participant inclusion criteria using forty-two participants (i.e. excluding only the two participants with excessive head-motion) were largely similar (Supplementary Fig. S2). None reported psychiatric or neurological disorders. All participants gave written informed consent and were paid for their participation. The protocol was approved by the Institutional Review Board of the Beijing MRI Center for Brain Research. All methods were performed in accordance with relevant named guidelines and regulations.

### Experimental design

Participants viewed two kinds of action videos (social interaction and object-manipulation), with both the agent and object held constant (a human figure and an arbitrary meaningless shape). Videos were made by a professional animation company and included actions performed by the same cartoon figure towards the meaningless shapes (see Fig. 2a for screenshot; all video stimuli were shown in Supplementary files). The actions correspond to two domains, with six actions in each type: social-communicative-actions (waving; saluting; bowing; kissing; clapping; greeting) and manipulation-actions (folding; tearing; overturning; rotating; pressing upper and lower; pressing left and right). The experiment also included a third navigation condition for other interests and were not considered for the main analyses. Each meaningless shape consisted of an outline and an interior shape (see exemplars in Fig. 2b). Six different interior meaningless shapes^49^ were combined with three different outline shapes (hexagon, circle, and square) to form the meaningless shapes. To verify the sociality of the social-communicative-actions, we collected sociality ratings on a 7-point scale (how likely these actions are directed at people, 1 = never, 7 = always) in an independent group of participants (*N* = 23, 16 females, mean age = 22.9). The social-communicative-action condition was indeed rated to be significantly more person-directed than the manipulation-action condition [mean ratings 6.04 ± 0.65 vs. 3.02 ± 1.55; *t*(22) = 9.104, *p* = 6.468 × 10^–9^]. The participants were instructed to remember the correspondence between action and shape. They were asked to report the action name associated with the presented shape and simulate the action after scanning. The three types of meaningless shapes (outlines) were counterbalanced across action types in a between-participant fashion (Fig. 2a), such that meaningless shapes included in the different action types were fully matched at the group level. Equal number of participants in each action-shape matching group. It should be noted that in the manipulation-action condition, the action induced shape to change form, as an intrinsic consequence of manipulation. These visual shape changes made the cumulative movements significantly higher in the manipulation-action than the social-communicative-action condition (sum of the amount of changes of the whole stimuli for each frame relative to the previous one; absolute value of pixel changes: *p* = 1.644 × 10^–7^). While this difference (i.e., form changes of meaningless shape in the manipulation-actions but not in the social-communicative-actions) did not correspond to any known domain differences between inanimate and animate objects, we further considered the potential effects of this confounding variable (amount of visual changes) on action domain effects by looking at another condition of no-interest where the amount of visual changes were even greater than the manipulation action condition (see “Results” section).

A long-block experimental design was employed, with 36 time points for each block. This allowed enough number of time points to calculate time series correlation without needing to concatenate different blocks from the same condition. This design is optimal to evaluate both regional activities and task-based FC^50–52^. The video-watching task included 4 runs. Each run consisted of a 10 s red fixation dot presented centrally, followed by 3 blocks from the different action conditions. Each block consisted of 24 trials from the same condition (i.e., each exemplar was repeated 4 times), followed by 10 s fixation. Each 3000-ms trial consisted of an action stimulus that lasted for 2000 ms, followed by 1000 ms of standing still (or running without turning in the navigation condition). The trial order was random and the block order was counterbalanced in a Latin square fashion across runs and participants. The experimental procedure was presented using Psychtoolbox (http://psychtoolbox.org/) implemented in MATLAB (https://www.mathworks.com/products/matlab.html).

### MRI data acquisition and preprocessing

The whole scanning session for each participant was about 95 min: (a) resting functional MRI scan; (b) meaningless shape viewing scan; (c) T1 functional scan; (d) video-watching scan (main experiment); (e) meaningless shape viewing scan; (f) diffusion tensor imaging. Data of procedure a, b, e, f were designed for another question and not analyzed herein. Whole scan data were acquired using a Siemens Trio Tim 3-T scanner at the Beijing MRI Center for Brain Research. T1-weighted three-dimensional magnetization-prepared rapid gradient echo images were obtained in the sagittal plane (repetition time (TR) = 2530 ms, echo time (TE) = 3.39 ms, flip angle = 7°, slice thickness = 1.3 mm, slice gap = 0.65 mm, slice in-place resolution = 1.3 × 1.0 mm^2^, field of view (FOV) = 256 × 256 mm^2^, slice number = 144). Functional images were acquired using an echo planar imaging sequence in the axial plane (TR = 2000 ms, TE = 30 ms, flip angle = 90°, slice thickness = 3.5 mm, slice gap = 0.7 mm, slice in-place resolution = 3.1 × 3.1 mm^2^, FOV** = **200 × 200 mm^2^, slice number = 33). The scanner was upgraded during our experiment. All parameters remain the same except for the slice number (it was changed to 32 for technical reasons). We had decent number of participants before (*N* = 16) and after (*N* = 20) the upgrade, and have looked at results separately, which had similar pattern to the combined (see Supplementary Fig. S3). We thus combined all participants as one group in the main analysis to improve power.

Functional images were preprocessed using Statistical Parametric Mapping (SPM12, http://www.fil.ion.ucl.ac.uk/spm). The first 5 volumes in each run were discarded. Three-dimensional head-motion correction was conducted with respect to the mean volume of each run. Two participants were excluded for excessive head motion (above 2 mm or 2°). No other participants exhibited excessive head motion (< 1.47 mm or 1.11°). For each participant, T1 images were co-registered to their mean functional images and were subsequently segmented. Functional images were normalized to the Montreal Neurological Institute (MNI) space using T1 image unified segmentation. After normalization, functional images were resampled to 3 × 3 × 3 mm^3^ and spatially smoothed with a 6 mm Full Width Half Maximum Gaussian filter. For FC analyses, the following preprocessing steps were additionally performed: linear trend removal, band-pass filtering (0.01–0.1 Hz), and regression of eight nuisance covariates (six rigid-body head-motion parameters, white matter signal, and cerebrospinal fluid signal). Global signal regression is controversial^53,54^ and often causes ‘negative’ correlations among brain regions. Therefore, we did connectivity analyses using data without global signal removal and repeated the analyses with global signal regression as validation analyses (see validation results in Supplementary Figs. S6 and S7; Supplementary Table S2). The residual time series with these nuisance covariates regression were used to do FC analyses.

### Identifying the domain-specific action perception systems: Social-communicative-actions versus manipulation-actions

First, we carried out whole-brain univariate analysis for the video-watching task to test whether social-communicative-actions and manipulation-actions would lead to differential brain activity. The whole-brain analyses were conducted using SPM12. In the first-level analysis, all preprocessed functional data were analyzed using a general linear model (GLM). We included nine predictors: the three video-watching experimental conditions and six motion parameters. The default value of the high-pass filter (128 s) was used to remove confounding influences on the BOLD signal, such as physiological noise from cardiac and respiratory cycles. Contrasts between social-communicative-actions and manipulation-actions, and between social- or manipulation-actions relative to baselines were built and computed for each participant. Then, in the second-level analysis, one-sample t-test analyses were applied to compare the mean activation across participants with zero (threshold set as voxel level *p* < 0.0001, cluster-extent FWE corrected *p* < 0.05). In this step, we used a gray matter mask that included voxels with a probability higher than 0.4 in the SPM5 gray matter template. All of the surface brain maps in the present study were visualized with the BrainNet Viewer^55^ (http://www.nitrc.org/projects/bnv/).

### FC analyses: communication pattern between the domain-specific action perception system and the ventral object perception regions

To explore how the domain-specific action perception systems communicate with the ventral object perception regions during action perception, we carried out task-state FC analyses. Specifically, we calculated the FC, seeding from the social- or manipulation-action perception systems, with ventral object perception regions during the two video conditions. We then applied repeated-measure ANOVAs to these FC measures. Figure 2c shows a flowchart of these analyses.

#### Region of interest (ROI) definition

Given that we did not have an independent localizer scan, we defined the social- and manipulation-action ROIs using a leave-one-participant out approach, so that the action perception systems definition data was independent from the FC analyses. We used N-1 participants to run the whole brain univariate contrast analyses (social-communicative-action perception vs. manipulation-action perception) and used the peak voxels to define action system ROIs for that remaining participant. Iterating this procedure 36 times defined the two sets of action system ROIs for each participant. We then used those peaks to form 3 mm radius sphere action perception ROIs (Fig. 2c; All peak coordinates presented in Supplementary Table S4). Three out of the 31 ROIs across all iterations were within VOTC and were excluded.

We considered ventral object perception regions in two ways: (1) In a ROI approach, we defined classical regions showing preference for social entities and manipulable objects/tools using the Neurosynth meta-analyses platform (https://neurosynth.org). Specifically, we searched for the terms “face” and “tool” separately in Neurosynth and retrieved the association threshold maps (896 studies including the word “face” and “115” studied including the word “tools”; default threshold at FDR corrected, *p* < 0.01). It should be noted that we also used “artifacts” or “manipulable objects” to search, but no results were found. We used “tools” rather than “objects” because the former is more specific to manipulation actions. These maps were resliced into the same voxel size with the functional images, i.e., 3 × 3 × 3 mm^3^. The strongest peaks within VOTC for “face” were in the bilateral lateral fusiform gyrus (i.e., fusiform face area, FFA), and for “tools” in the left lateral occipitotemporal cortex (LOTC; Supplementary Fig. S4; Table S4). These peaks were extracted and used to form sphere ROIs of 3 mm radius. A small significant cluster for tool in the medial fusiform gyrus (medFG) was further included given its relevance highlighted in previous literature^12,29,56^; (2) in a data-driven approach, we used a whole VOTC mask, obtained using previous dataset in our lab, to test the dynamic FC patterns of dorsal action perception system and ventral object perception system. It was defined by combining functional and anatomical localization, including regions that were activated during an object picture perception task within the occipitotemporal cortex (*z* coordinate below 10; see Wang et al.^57^, procedure following Kriegeskorte et al.^58^).

#### FC computation

The following steps were carried out in each participant separately*.* First, the task-state residual time series of each run was segmented into separate conditions as follows: for each block in each run, the first 4 volumes (8 s) were discarded, and 2 volumes (4 s) of the 10 s fixation were included to account for the hemodynamic delay. Within each ROI sphere of the action systems, the residual time series of all voxels were averaged. Then, we computed the FCs seeding from each social- or manipulation-action perception ROI sphere with each Neurosynth-defined ventral object domain ROI (in the ROI analysis), or each voxel in the VOTC mask (in the Whole VOTC mask analysis), under the two action perception conditions separately. The correlation coefficients were then Fisher-*z* transformed and averaged across all ROIs within an action system, across all runs within an experiment condition, to yield four measures for each ventral ROI or voxel (Fig. 2c): FC with the social-communicative-action system in the social-communicative-action condition, FC with the social-communicative-action system in the manipulation action condition, FC with the manipulation-action system in the social-communicative-action condition, FC with the manipulation-action system in the manipulation-action condition. Note that in the main analysis, multiple ROIs in the same action systems were averaged together for the ANOVA analysis. We also report the FC results between each separate action perception ROI sphere and each Neurosynth-defined object perception ROI (Supplementary Fig. S9).

#### ANOVA

For Neurosynth-defined ventral classical object perception ROIs, we applied repeated-measure ANOVAs using SPSS Statistics 20 (https://www.ibm.com/cn-zh/analytics/spss-statistics-software) to test whether social- and manipulation-action perception systems interact with face and tool perception regions in a domain-specific way. We then applied repeated-measure ANOVAs using SPM12 to identify brain regions showing significant main effects of action brain system/action perception condition or interaction effects between the action brain system and action perception condition within whole VOTC mask (threshold set as voxel level *p* < 0.001, cluster-extent FWE corrected *p* < 0.05). For regions showing significant interaction effects, subsequent comparisons were performed (two-tailed paired-sample *t*-tests) to test the specific connection patterns among the four FC conditions (all Bonferroni-corrected based on four comparisons of simple effects).

### Relationship between VOTC’s FC with dorsal action perception systems and VOTC’s local activation strength

To examine whether dorsal action systems could affect the VOTC activation through functional coupling when object-domain information had been well-controlled, we calculated the correlation between FC strength with a specific action system and activation strength in each action perception conditions across VOTC voxels. First, for each VOTC voxel we calculated its average FC strength across all participants for each of the four measures (with two action systems in two conditions). Then, for each VOTC voxel we calculated the average activation strength across participants for social-communicative-action and manipulation-action perception conditions separately. Then we computed the Pearson correlation coefficient between the domain-specific FC strength (i.e., connected with one of the action systems relative to the other one in the same action perception condition) and the domain-specific activation strength (i.e., in one action condition relative to the other) across all of the VOTC voxels.

## Supplementary information

Supplementary Information.

Supplementary videos.

## Data Availability

The data that support the results of the present study are available from the corresponding author upon reasonable request.
