# Supplementary material for: Domain-specific functional coupling between dorsal and ventral systems during action perception
Source: Sci Rep. 2020 Dec 3;10:21200. doi: 10.1038/s41598-020-78276-4 (PMC7713359; doi:10.1038/s41598-020-78276-4)

Supplementary information for

Waving or folding? Domain-specific functional coupling between action and ventral object perceptual systems during action perception

Huichao Yang, Chenxi He, Zaizhu Han, Yanchao Bi.

Participant exclusion

Forty-four individuals who were assigned to three different action-shape matching groups (Supplementary Table S1, group 1 and 2 had 15 participants and group 3 had 14 participants) participated our experiment. Firstly, we excluded two participants with extra head-motion. They were both from the group 3. In order to make the meaningless shapes included in different action types fully matched at group level, we need to exclude three participants from group 1 and group 2, respectively. Besides, we balanced the block order in a Latin square fashion across runs and participants (a-e represent six different condition/block orders in Supplementary Table S1). Accordingly, we excluded three participants with relative lower behavior scores within group 1 and 2. Behavior score was the recall accuracy (i.e., the percentage of correct recall action-shape correspondence number out of the total number) after scanning. After exclusion, each block order was presented with equal times across thirty-six participants who belong to the three action-shape matching groups with equal number.

The exclusion criteria were relatively strict in order to counterbalance the meaningless shapes between difference action conditions and make the experiment more prudent. Results of using more liberal participant inclusion criteria using forty-two participants (i.e. excluding only the two participants with excessive head-motion) were largely similar (Supplementary Figure S2; threshold set as the same with the main text, i.e., voxel level $p < .0001$, cluster-extent FWE corrected $p < 0.05$).

Scanner upgrade

As mentioned in the main text, the scanner was upgraded during our experiment. All parameters remain the same except for the slice number (it was changed to 32 for technical reasons). The whole-brain univariate results of data before and after scanner upgrading were presented in Supplementary Fig. S3 (threshold set as $p < .001$, cluster size > 10 resampled voxels, uncorrected). Fifteen participants were included in the pre-upgrade analyses and eighteen were included in the post-upgrade analyses. Three participants with lower behavior score were excluded to balance the action-shape matching groups between-subject as in the main text (one for pre-upgrade and two for post-upgrade; sub-03, sub-35, sub-43 in Supplementary Table S1). The result patterns were stable between pre- and post-

upgrade and similar with the main results of the whole thirty-six participants (see Figure 1 in the main text).

Validation results using data with global signal regression in preprocessing

We used data preprocessed with global signal regression to repeat the ANOVA over the Neurosynth-defined ventral object perception ROI-based and VOTC-based FC maps. Only those results showing statistical significance are reported.

ROI analyses results. FC patterns of bilateral FFA and left LOTC were replicated (Supplementary Figure S6) but the effects were weaker than primary results. The interaction effects of bilateral FFA were significant if we grouped them together (i.e., computing average value of bilateral FFA: $F(35) = 4.343$, p = .045), but the effects was not significant when separately. These regions showed same FC patterns with primary results: They significantly increased their FCs with the social-communicative-action system during social-communicative-action perception condition compared to the manipulation-action perception condition (green bar > blue bar for social-communicative-action system in Supplementary Fig. S6a&b; left: $t(35) = 2.585$, uncorrected $p = 0.014$; right: $t(35) = 2.318$, uncorrected $p = 0.026$; grouped bilateral: $t(35) = 3.308$, adjusted $p = .009$). And their connection with the social-communicative-action system was also significantly stronger than with the manipulation-action system in the social-communicative-action perception condition (two green bars in Supplementary Fig. S6a&b; left: $t(35) = 2.766$, adjusted $p = 0.036$; right: $t(35) = 2.898$, adjusted $p = 0.026$; grouped bilateral: $t(35) = 3.178$, adjusted $p = .012$). The left LOTC showed significant interaction effects between action perception system and action viewing conditions ($F(35) = 7.057$, $p = .012$). It showed stronger connections with the manipulation-action system in the manipulation-action perception condition than in the social-communicative-action perception condition (Supplementary Figure S6c; $t(35) = 2.282$, uncorrected $p = .029$). For the left medFG tool-preferring ROI, we found it was more strongly connected with the manipulation-action system in both action perception conditions (i.e., significant main effects of action system, $F(35) = 7.214$, $p = .011$; Supplementary Figure S6d). But this pattern was not found in the primary results.

Whole VOTC mask analyses results. We reported clusters significant (voxel level $p < .001$, cluster-

extent FWE $p < 0.05$; either main or interaction effects) in both primary and validation analyses (i.e. with or without global signal regression) as in the main text. One cluster encompassing the right inferior temporal gyrus (ITG) and fusiform gyrus (FG) were obtained showing significant interaction (Supplementary Figure S7a and Table S2): It connected with the manipulation-action system stronger in the manipulation-action condition than social-communicative-action condition ($t(35) = 4.573$, adjusted $p = 2.313 \times 10^{-4}$), and with the social-communicative-action system were stronger in the social-communicative-action condition than in the manipulation-action condition ($t(35) = 3.095$, adjusted $p = .015$). The right superior temporal pole (sTP) showed a significant main effect of action system without interaction with action condition: They had stronger connections with the social-communicative-action system than with the manipulation-action system (Supplementary Figure S7c, Table S2).

FC patterns between each separate action perception ROI and each Neurosynth-defined ventral object perception ROI

To test whether the FC patterns were stable across different ROIs within the action perception systems, we replicated the Neurosynth-defined ROI-based FC analyses by computing FCs between each object perception ROI and each action perception ROI separately (instead of averaging across ROIs within same action system in the primary analyses). To do this, we need to defined ROIs of action perception systems in group-level (rather than leave-one-participant out methods in the primary analyses) and thus ensure that ROIs were consistent across all of the participants. Specifically, peak coordinates were extracted from the primary univariate maps (Figure 1 and Table 1) and were used to form sphere ROIs of 3 mm radius (the ROIs were shown in Supplementary Fig. S9a). Other procedures were identical with the primary processing.

First, results of each Neurosynth-defined ROI were largely replicated across different methods of action perception ROI definitions (i.e., leave-one-participant out, group-level). Bilateral FFA ($F(35)$s ≥ 9.027, $p \leq .005$) and left LOTC ($F(35) = 9.170$, $p = .005$) showed significant interaction effects. Neither main effects nor interaction effects were found for the left medFG (ps $\geq .097$). Bilateral FFA showed a domain-specific connection pattern with the social-communicative-action system and left

LOTC showed a domain-specific connection pattern with the manipulation-action system (see specific patterns in the upper panel of Supplementary Fig. S9b-e).

Second, the FC patterns of each object perception ROI with each action perception ROI were similar with the FC patterns averaged across ROIs within same action perception system (Supplementary Figure S9). Take the right FFA as an example, it connected with the social-communicative-action system stronger in the social-communicative-action perception than in the manipulation-action perception condition ($t(35) = 2.899$, adjusted $p = 0.026$), but no significant differences were found for FC with the manipulation-action system between different action perception conditions ($p = 0.316$). FC patterns between the right FFA and each action perception ROI were similar: it connected with each social-communicative-action perception ROI stronger in social-communicative-action perception than in manipulation-action perception (even not all of them were significant); no significant differences were found for manipulation-action perception ROIs between different action conditions. The other ROIs were analogous to this one.

Supplementary Table S1. Participant exclusion information.

Participant	Head-motion （"x" above 2 mm or 2°; "√" no excessive head-motion）	Group number	Block order for each run	Behavioral score	Final inclusion ("×" excluded; "√" included)
sub-21	√	1	cabe	0.00	×
sub-24	√	1	efda	0.00	×
sub-01	√	1	cabe	0.06	×
sub-03	√	1	cabf	0.06	√
sub-22	√	1	efdc	0.33	√
sub-05	√	1	cabd	0.50	√
sub-26	√	1	efdb	0.61	√
sub-27	√	1	cabe	0.61	√
sub-28	√	1	efdc	0.78	√
sub-04	√	1	efda	0.83	√
sub-19	√	1	cabe	0.83	√
sub-02	√	1	efdc	0.89	√
sub-06	√	1	efdb	0.94	√
sub-23	√	1	cabf	1.00	√
sub-25	√	1	cabd	1.00	√
sub-35	√	2	bcaf	0.17	√
sub-31	√	2	bcad	0.22	×
sub-09	√	2	bcad	0.44	×
sub-11	√	2	bcae	0.44	√
sub-33	√	2	bcae	0.50	√
sub-07	√	2	bcaf	0.67	√
sub-08	√	2	fdeb	0.67	×
sub-36	√	2	fdeb	0.67	√
sub-10	√	2	fdec	0.72	√

Participant	Head-motion<br>（“x” above 2 mm or 2°;<br>“√” no excessive head-motion）	Group number	Block order for<br>each run	Behavioral score	Final inclusion<br>(“×” excluded;<br>“√” included)
sub-29	√	2	bcaf	0.78	√
sub-20	√	2	bcad	0.83	√
sub-32	√	2	fdec	0.89	√
sub-30	√	2	fdeb	0.94	√
sub-34	√	2	fdea	0.94	√
sub-12	√	2	fdea	1.00	√
sub-14	√	3	defa	0.28	√
sub-17	×	3	abcf	0.33	×
sub-18	√	3	defc	0.61	√
sub-43	√	3	abcd	0.75	√
sub-44	√	3	defa	0.78	√
sub-40	√	3	defb	0.83	√
sub-42	×	3	defc	0.83	×
sub-39	√	3	abce	0.89	√
sub-41	√	3	abcf	0.94	√
sub-13	√	3	abcd	1.00	√
sub-15	√	3	abce	1.00	√
sub-16	√	3	defb	1.00	√
sub-37	√	3	abcd	1.00	√
sub-38	√	3	defa	1.00	√

Note: the participants were ordered by Group number and Behavioral accuracy; a-e represent six different block orders.

Supplementary Table S2. Validation results of VOTC-based ANOVA using data with global signal regression

Cluster label	Anatomical regions of the cluster's peak voxel (other including regions)	MNI coordinates of peak voxel (mm) x	y	z	F	Cluster size
Clusters showing significant interaction effects						
1	right ITG (FG)	51	-63	-15	25.19	53
2	left Lingual	-21	-75	-9	24.34	40
Clusters showing significant main effect of action systems						
1	right sTP	48	18	-21	45.55	43
2	left MTG (MOG)	-57	-66	3	43.64	75

Threshold: voxel level $p < .001$, cluster-extent FWE corrected $p < 0.05$; Abbreviations: ITG = inferior temporal gyrus; FG = fusiform gyrus; sTP = superior temporal pole; MTG = middle temporal gyrus; MOG = middle occipital gyrus.

Supplementary Table S3. Complete results of VOTC-based ANOVA for primary analyses using data without global signal regression.

Cluster label	Anatomical regions of the cluster's peak voxel (other including regions)	MNI coordinates of peak voxel (mm) x	y	z	F	Cluster size
Clusters showing significant interaction effects						
1	right ITG (FG/IOG/MTG)	54	-66	-12	32.82	270
2	left FG (ITG/IOG)	-42	-57	-21	23.24	87
3	left pMTG	-48	-57	3	18.94	20
Clusters showing significant main effect of action systems						
1	right sTP	51	18	-18	54.77	59
2	left sTP	-48	12	-18	35.62	20
3	right Lingual	24	-63	-6	26.58	74

Threshold: voxel level $p < .001$, cluster-extent FWE corrected $p < 0.05$; Abbreviations: ITG = inferior temporal gyrus; FG = fusiform gyrus; IOG = inferior occipital gyrus; MTG = middle temporal gyrus; pMTG = posterior middle temporal gyrus; sTP = superior temporal pole. Note: only clusters significant (voxel level $p < .001$, cluster-extent FWE $p < 0.05$) in both primary and validation analyses were shown in the main text (see Table 2).

Supplementary Table S4. Coordinates of action perception and Neusynth-defined ventral object perception ROIs

	x	y	z	number of participants using the peak
MA perception vs. SA perception	63	-18	33	1
	60	-18	33	35
	54	9	24	25
	27	-9	63	35
	24	-12	60	1
	**-9**	**-90**	**-9**	1
	**-15**	**-84**	**-6**	2
	-24	-12	54	1
	-36	-45	60	23
	-39	-6	12	17
	-39	-9	15	1
	-39	-3	12	1
	-39	-3	9	1
	-39	-42	57	2
	-39	-45	57	1
	-42	-6	12	9
	-54	-30	42	7
	-54	6	33	1
	-54	6	36	22
	**-54**	**-63**	**-12**	1
	-57	-27	39	1
	-60	12	12	2
	-60	-18	39	1
	-60	-15	30	1
SA perception vs. MA perception	42	3	45	36

	x	y	z	number of participants using the peak
	57	-42	15	34
	57	-39	15	2
	-57	-48	12	5
	-57	-45	9	1
	-66	-42	6	2
	-66	-42	9	28
Neurosynth ventral object perception ROI – right FFA	42	-48	-21	/
Neurosynth ventral object perception ROI – left FFA	-39	-51	-21	/
Neurosynth ventral object perception ROI – left LOTC	-51	-60	-3	/
Neurosynth ventral object perception ROI – left medFG	-33	-30	-24	/

Coordinates marked in bold were excluded for locating in VOTC mask. Abbreviations: SA = social-communicative-actions; MA = manipulation-actions;

Figure legends

Supplementary Figure S1. Whole brain univariate analysis results of social- and manipulation-action perception relative to baseline. **a**, Social-communicative-action activation relative to the baseline. **b**, Manipulation-action activation relative to the baseline. **c-d**, Conjunction (overlap) of social- and manipulation-action conditions relative to the baseline.

Supplementary Figure S2. Whole-brain univariate analysis results of social- or manipulation-action-specific activations using data of 42 participants. **a**, Social-communicative-action specific activation relative to the manipulation-action condition. **b**, Manipulation-action specific activation relative to the social-communicative-action condition. Threshold: voxel level $p < .0001$, cluster-extent FWE corrected $p < 0.05$.

Supplementary Figure S3. Whole-brain univariate analysis results of social- or manipulation-action-specific activations using data acquired before and after the scanner upgrade. **a**, Results of before-upgrade. **b**, Results of after-upgrade. Threshold: voxel level $p < .001$, cluster-extent $k > 10$, uncorrected.

Supplementary Figure S4. Association threshold maps from Neurosynth meta-analyses (FDR corrected, $p < .01$; https://neurosynth.org) that were used to define ventral object-perception ROIs, i.e., bilateral fusiform face area (FFA), left lateral occipitotemporal cortex (LOTC) and left medial fusiform gyrus (medFG). **a**, Brain maps of Neurosynth meta-analyses across 896 studies that include the word "face". **b**, Brain maps of Neurosynth meta-analyses across 115 studies that include the word "tools".

Supplementary Figure S5. Validation results for the potential confounding of amount of movements. **a**, Activation strength's comparison between navigation and manipulation-actions in ROIs showing manipulation specific-activations (relative to social-communicative-actions). Red asterisks indicate significant difference between the two conditions ($p < .05$). **b-e**, FC results between the action perception systems and the Neurosynth ventral object perception regions for tools and faces (see Figure 3), with the manipulation-action perception systems excluding the right inferior

frontal gyrus/precentral cluster (MNI peak coordinates: 54, 9, 24). We applied tests of simple effects either within the same action system or within the same action condition for ROIs showing significant interaction effects; lines above bars indicate significant difference between the two bars (black: adjusted $p < .05$; gray: uncorrected $p < .05$). FFA = face fusiform area; LOTC = lateral occipitotemporal cortex; medFG = medial fusiform gyrus. MA = manipulation-actions. SA = social-communicative-actions.

Supplementary Figure S6. Validation results of Neurosynth-defined ventral ROI-based ANOVA using data with global signal regression in preprocessing. **a-b**, FC patterns of the face-preferring left and right fusiform face area (FFA): enhanced connection with the social-communicative-action system in social-communicative-action perception. **c**, FC patterns of the tool-preferring left lateral occipitotemporal cortex (LOTC): enhanced connection with the manipulation-action system in manipulation-action perception. **d**, FC patterns of the tool-preferring left medial fusiform gyrus (medFG): connected with manipulation-action system stronger in both action condition. We applied tests of simple effects either within the same action system or within the same action condition for ROIs showing significant interaction effects; lines above bars indicate significant difference between the two bars (black: adjusted $p < .05$; gray: uncorrected $p < .05$). Asterisks indicates significant main effect ($p < 0.05$). MA = manipulation-actions. SA = social-communicative-actions.

Supplementary Figure S7. Validation results of VOTC-based ANOVA using data with global signal regression in preprocessing (threshold: voxel level $p < .001$, cluster-extent FWE $p<0.05$). **a-b**, FC patterns of clusters showing significant interaction effects between action systems and action condition (red in the brain). We applied tests of simple effect either within the same action system or within the same action condition; black lines above bars indicate significant difference between the two bars (adjusted $p < .05$). **c-d**, FC pattern of clusters showing significant main effect of action system without interaction with action conditions (green in the brain). ITG = inferior temporal gyrus; FG = fusiform gyrus; sTP = superior temporal pole. MA = manipulation-actions. SA = social-communicative-actions.

Supplementary Figure S8. Complete results of VOTC-based ANOVA for primary analyses (i.e.,

using data without global signal regression in preprocessing; threshold: voxel level $p < .001$, cluster-extent FWE $p<0.05$). **a-c**, FC patterns of clusters showing significant interaction effects between action systems and action condition (red in the brain). We applied tests of simple effects either within the same action system or within the same action condition; black lines above bars indicate significant difference between the two bars (adjusted $p < .05$). **d-f**, FC pattern of clusters showing significant main effect of action system without interaction with action conditions (green in the brain). Note: only clusters significant (voxel level $p < .001$, cluster-extent FWE $p < 0.05$) in both primary and validation (data with global signal regression) analyses were shown in the main text (see Figure 4 and Table 2). pMTG = posterior middle temporal gyrus; FG = fusiform gyrus, ITG = inferior temporal gyrus; IOG = inferior occipital gyrus; sTP = superior temporal pole. MA = manipulation-actions. SA = social-communicative-actions.

Supplementary Figure S9. FC patterns between each separate action perception ROI and each Neurosynth-defined ventral object perception ROI using data without global signal regression. **a**, Neurosynth-defined ventral object perception ROIs and social-/manipulation-action system ROIs. Note that the action perception ROIs were defined in group-level using univariate contrast analysis results of 36 participants (rather than leave-one-participant in the primary analyses) to make the action perception ROIs consistent across participants. MA = manipulation-actions; SA = social-communicative-actions. FFA = fusiform face area; LOTC = lateral occipitotemporal cortex; medFG = medial fusiform gyrus. **b-e**, Upper panel: FC patterns of each Neurosynth-defined ventral object perception ROI with the two action systems (averaged across all ROIs within same action system) under two action conditions. Lower panel: FC patterns between each Neurosynth-defined ventral object perception ROI and each ROI of the two action perception systems under two action conditions separately. We applied tests of simple effects either within the same action system or within the same action condition for object ROIs showing significant interaction effects: lines above bars indicate significant difference between the two bars (black: adjusted $p < .05$; gray: uncorrected $p < .05$). We applied paired t-test FCs with each action perception ROI under two action conditions; red asterisks indicate significant difference between the two conditions ($p < .05$).

Supplementary Figure S1.

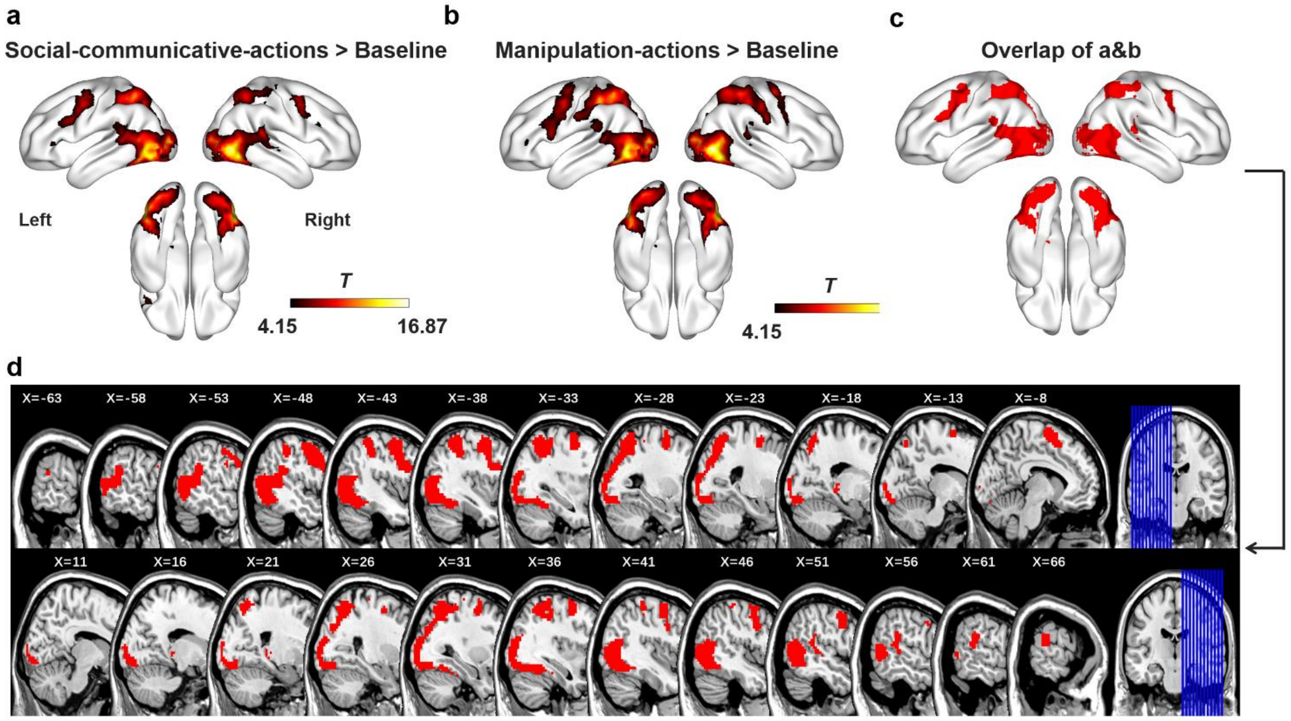

Supplementary Figure S2.

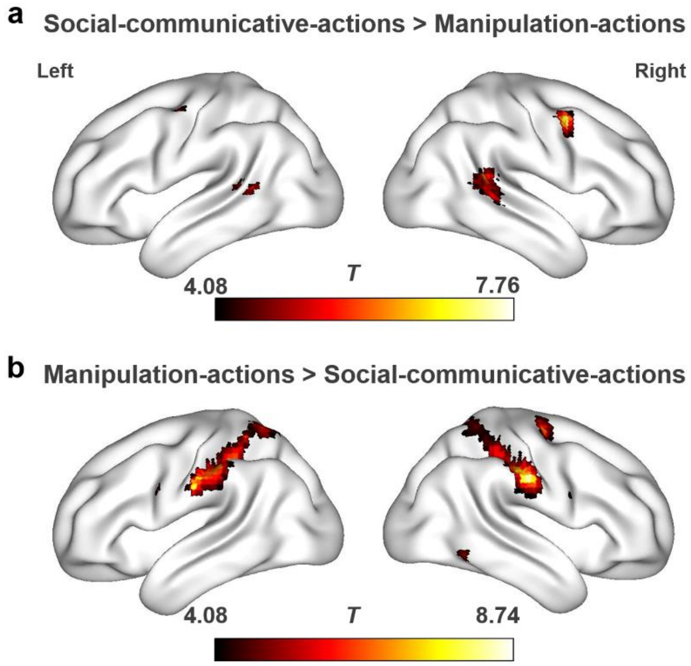

Supplementary Figure S3.

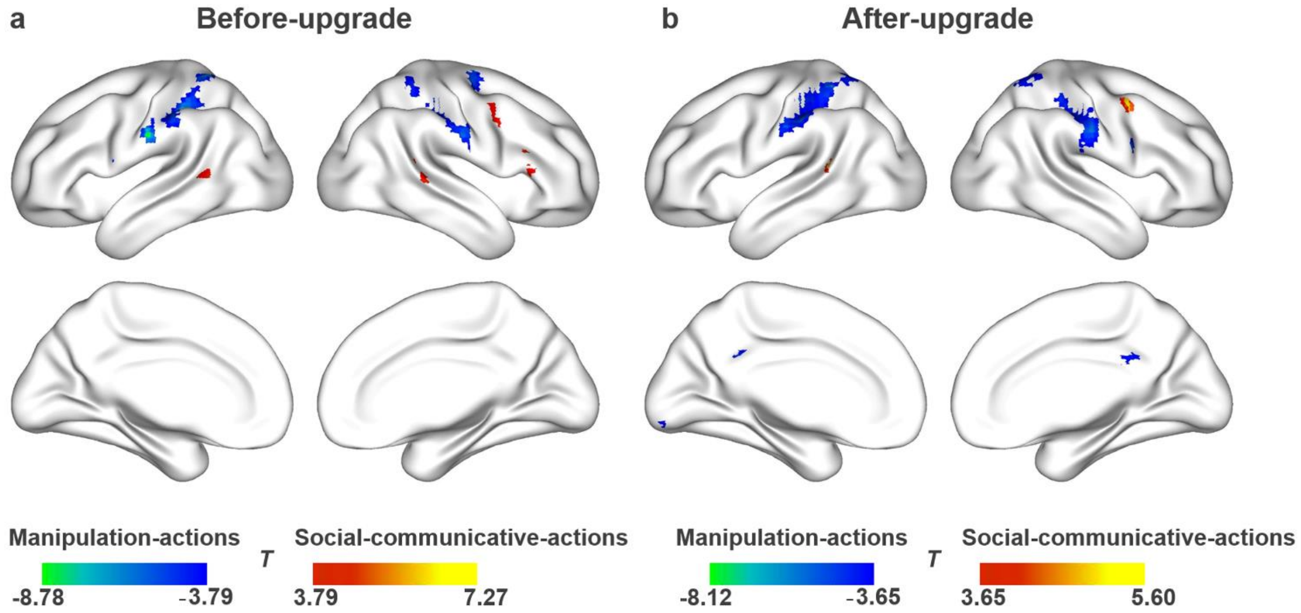

Supplementary Figure S4.

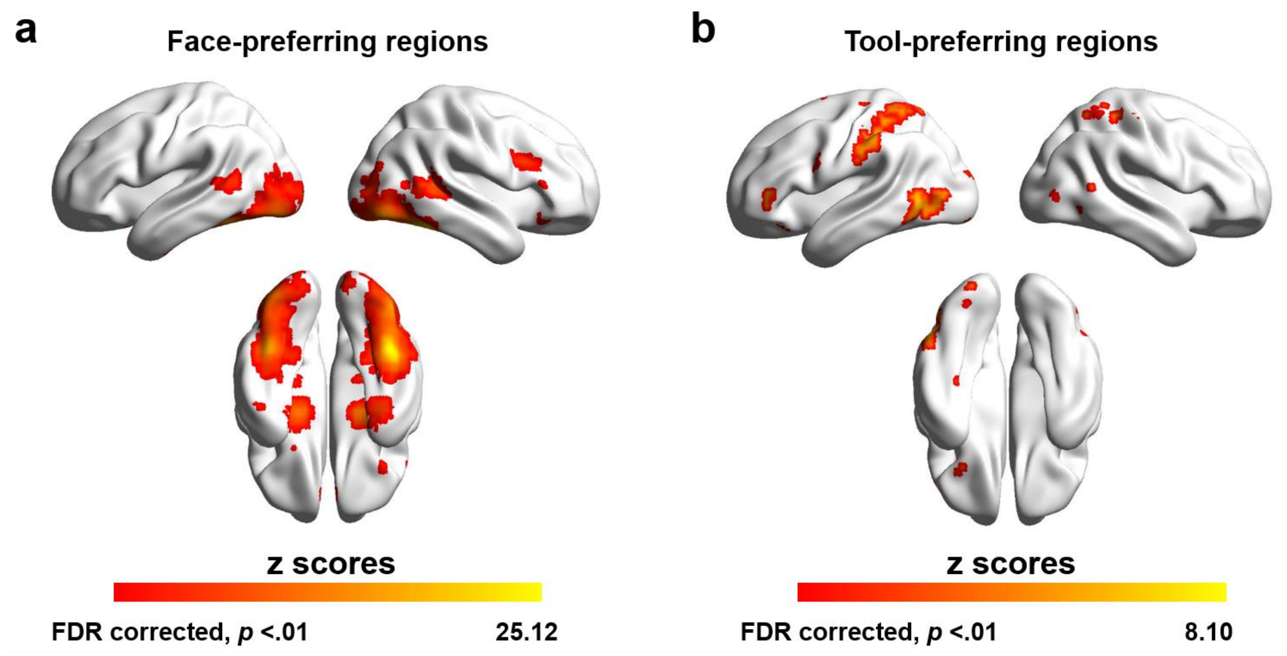

Supplementary Figure S5.

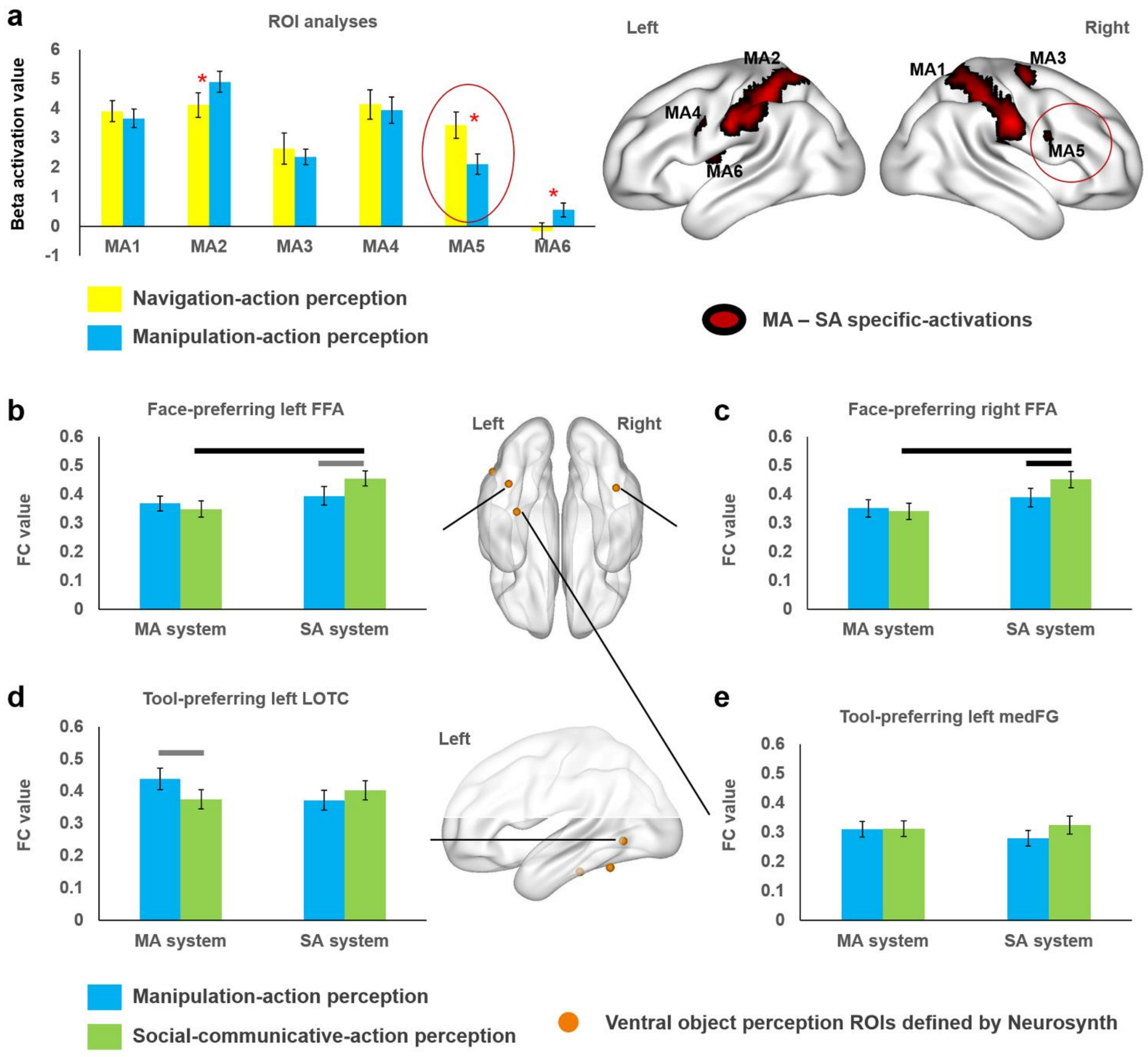

Supplementary Figure S6.

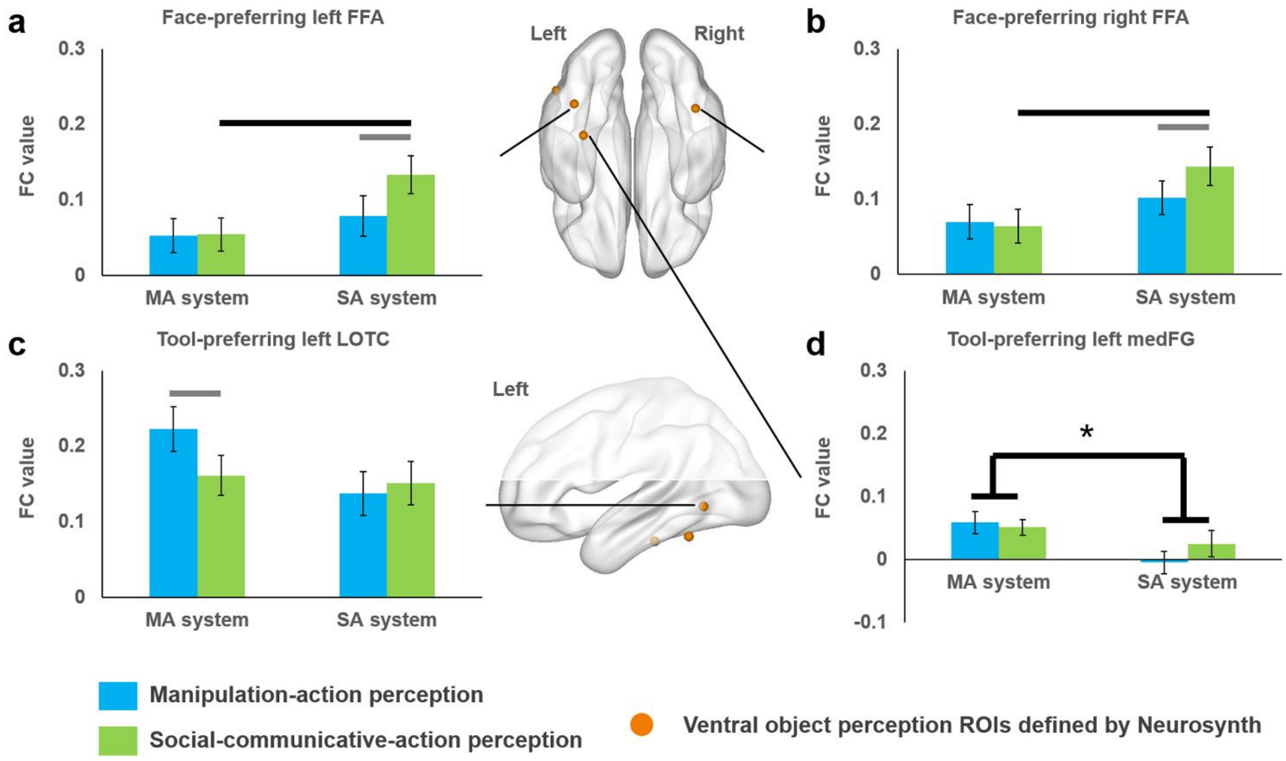

Supplementary Figure S7.

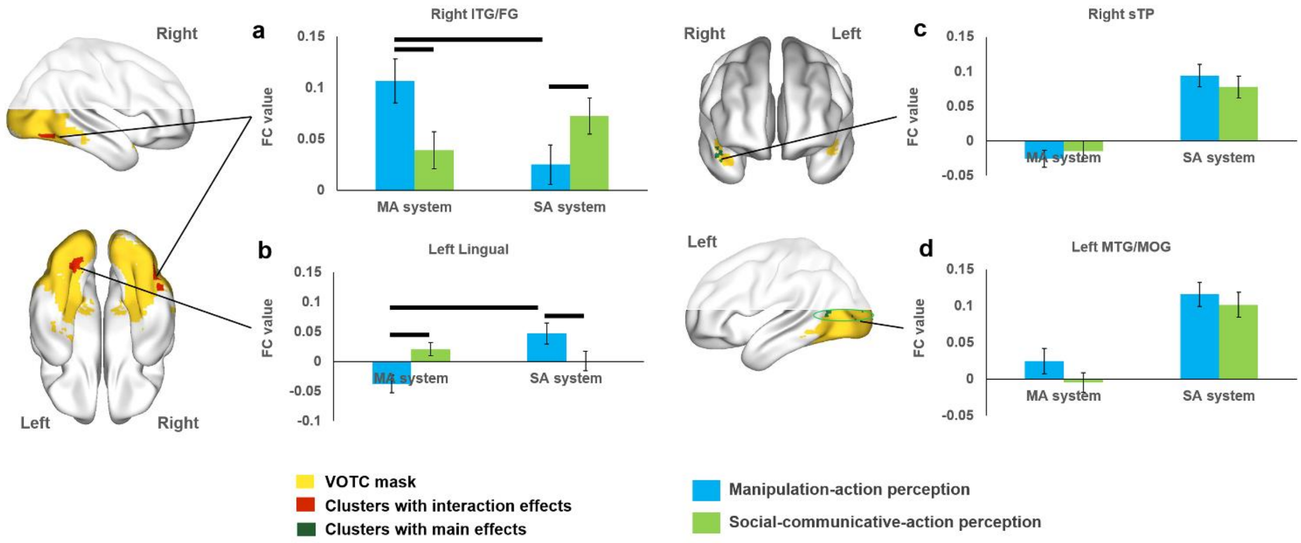

Supplementary Figure S8.

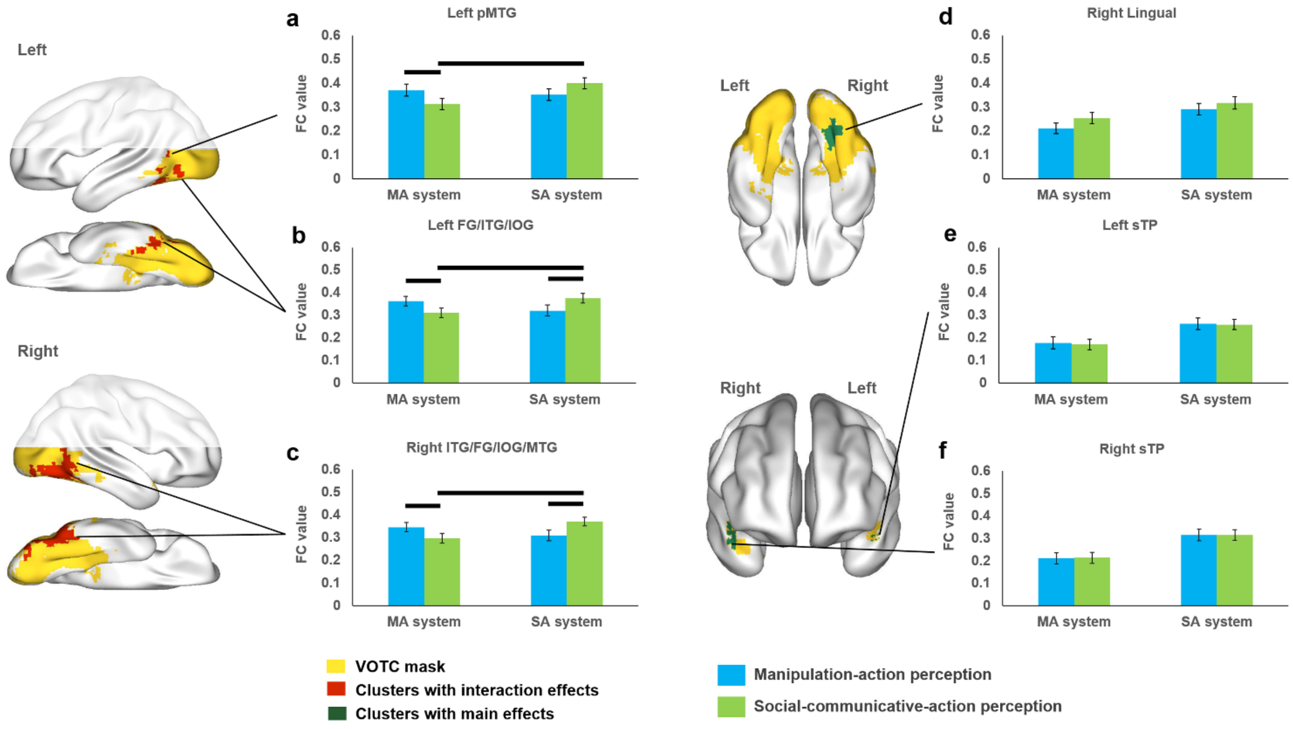

Supplementary Figure S9.

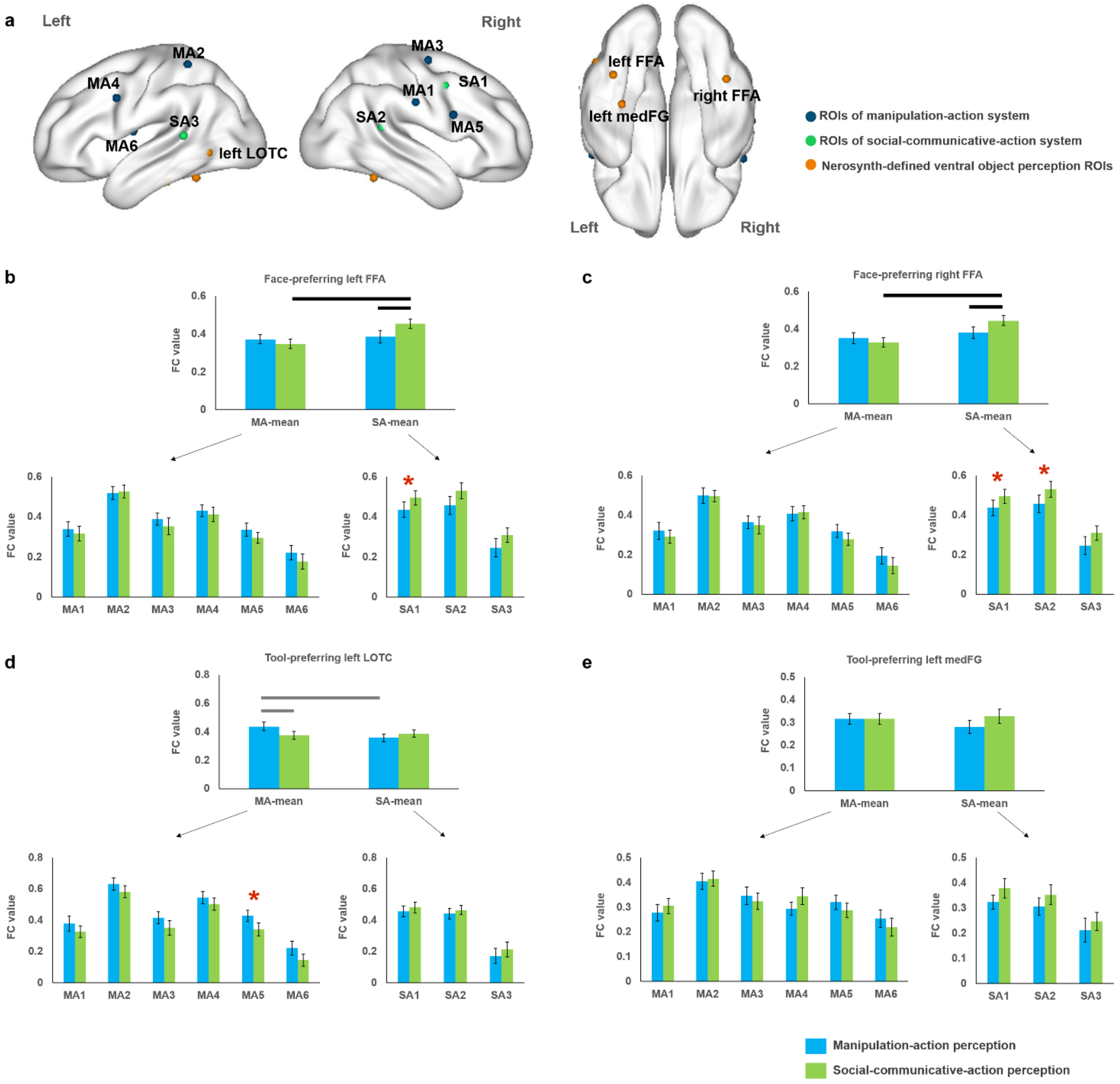

Supplement: Supplementary file 1 — Supplementary Information. [file 41598_2020_78276_MOESM1_ESM.pdf]
